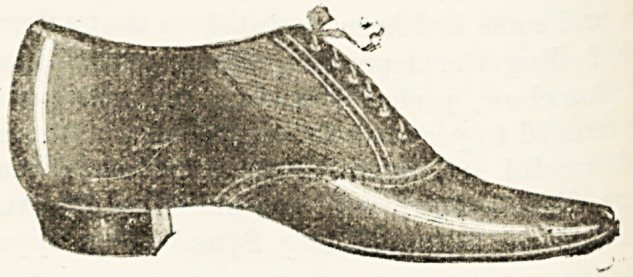# "The Hospital" Nursing Mirror

**Published:** 1899-12-30

**Authors:** 


"The Hospital" Nursing Mirror.

Being the Nursing Section of "The Hospital."

[Contributions for this Section of "The Hospital" should be addressed to the Editor, The Hospital, 28 & 29, Southampton Street, Strand, London, W.C., and should have the word "Nursing" plainly written in left-hand top corner of the envelope.]

### Notes on News from the Nursing World.

### THE NURSES OF THE ARMY RESERVE.

The nurses of the Army Reserve, who left Southampton on Saturday in the "Dunottar Castle" for the seat of war were Miss F. Bell, Miss A. Davidson, Miss L. M. Tippetts, and Miss N. Strangman. Miss Bell was trained at the Great Northern Hospital, London, and has been doing private nursing this summer. Miss Davidson was trained at the Temperance Hospital and the Samaritan Free Hospital, London. Her last appointment was at the Dublin Military Hospital, where she has been getting some experience in army nursing. Miss Tippetts was trained at Guy's, and has since been charge nurse in the operation theatre, and night sister with sole charge of the theatre for all night operations. Miss Strangman is Sister Victoria at St. Thomas's Hospital. We understand that among the further four nurses who start at the end of this week are Sister Bindloso, of St. Mary's Hospital, who has been eleven years in that institution; and Miss Herring, who was nearly six years nurse at the Devon and Somerset Hospital, and is now on duty at the Dover Military Hospital.

### NURSING AT THE FRONT.

The War Office authorities are said to have intimated that, "if they were to accept all nurses who had volunteered, there would not only be a nurse for every wounded soldier, but one apiece for every wounded Boer as well." Some of the volunteers who have been rejected may not be aware of the perils of nursing at the front. A nurse, who went to Ladysmith on October 28th, and afterwards accompanied the wounded to Durban, has vividly described a few of her experiences:—

"We were operating, amputating all Tuesday night, and they were dying all around us. I washed twenty men all alone. Some were put under the X Rays for the bullets. I, with many other nurses, including the Netley sisters, had many narrow escapes. During Monday the enemy kept shelling the town, and a shell fell right at Nurse M.'s and my feet. On Wednesday the twenty-four hours' truce was over which was granted by General White to the enemy to bury their dead and attend to the wounded. We were all up and ready for the Boers to shell the town, and no one who was not there can ever imagine what we felt like when 'Long Tom' began to fire, and our big guns were not in position then."

As she could not return to Ladysmith, she had official orders to remain at Durban for the present, and she says that all trained nurses everywhere have been commanded for active service, if necessary, by the Government. Moreover, there is no question of earning money. "Everybody has to give his or her services," and the nurses who are not nursing are engaged in making linen bandages, padding, splints, cotton-wool, and jackets for the Army Department, who give out so much for nurses to do for them.

### THE IMPERIAL NURSING STAFF AT LADYSMITH.

It is stated that there are ten nurses at Ladysmith working under the direction of the Army Medical Corps. Sister Dowse of the Army Nursing Service, is superintendent, and Mrs. Ludlow is in charge of the ward. Mrs. Ludlow, who is the wife of Major Ludlow, will be remembered by many of our readers as Miss Barton, matron at the Royal Free Hospital in Gray's Inn Road. She gave up nursing when she was married, but has resumed it for the time being, having accompanied her husband to the seat of the war. Three nurses from the Johannesburg Hospital are engaged at Ladysmith.

### SIR WILLIAM MacCORMAC AND THE NURSES AT WYNBERG.

Some very complimentary things are said of the nurses at Wynberg by Sir William MacCormac, who has paid a visit to the general hospital. The wards he found gay with spring flowers on the tables and at the bedsides; and in that, as in many other ways, he saw "the delightful evidence of woman's presence." The four nurses he describes as "bright and charming." "One of them," he says, "smilingly told me that there was simply nothing that she could not have for the patients for asking, and that the townspeople are profuse in their gifts of every kind for the comfort and pleasure of the patients." It is satisfactory to learn from Sir William that the sanitary arrangements are very complete, and that nurses and patients alike are so well cared for.

### THE NURSES ON THE PRINCESS CHRISTIAN HOSPITAL TRAIN.

Miss Jane Creighton, one of the nurses who accompanies the Princess Christian Hospital Train to the front, was trained at Guy's Hospital, becoming staff nurse in June, 1897. Since September, 1897, she has been sister in the same institution. Both Miss Creighton and Miss Jones, the other nurse, were personally selected by Princess Christian.

### PRINCESS SALM SALM AND THE BOERS.

The Princess Salm Salm, of whose romantic career an account was given in these pages on July 8th, has undertaken the superintendence of the nursing on the Boer side. Her husband, Mr. Charles Heneage, writing from Fulstow Hall, Louth, maintains that her employment is in our interests for a number of reasons. Thus, he says, that "our own sick and wounded in the hands of the enemy will be skilfully and properly nursed," and that "the horrors of war will be mitigated, because the Boers will be anxious not to do anything in the presence of the Princess Salm Salm which might injure their cause as regards public opinion with special reference to those natives which sympathise with the Boers." We hope, at any rate, that the wounded Boers, as well as our own wounded, will benefit by the presence among them of the Princess whose experience as nurse among the wounded in the Franco-German War should be very valuable.

### OUR CLOTHING DISTRIBUTION.

The results of our clothing distribution have been most successful this year. Our kind contributors responded so generously to our appeal that we were able to send off no fewer than 12 large parcels. In accordance with our custom, we tried to select the hospitals in poor districts. The institutions selected were: Charing Cross Hospital, King's College Hospital, the Royal Hospital for Children and Women, the Metropolitan Hospital (Kingsland Road), the Middlesex Hospital, the West London Hospital, the Marylebone Infirmary, the East London Hospital for Sick Children, the East-end Mothers' Home, the Great Northern Central Hospital, and the West Ham Hospital. Eleven hospitals were thus benefited by the kind efforts of our readers, the twelfth parcel of toys accompanying that of clothing to the East London Hospital for Sick Children.

### THE NEW NURSES' HOME AT EPSOM.

The Epsom Board of Guardians and the nurses under them are to be congratulated upon their new nurses' home, which was opened the other day without ceremony. There is nothing conventional about the building. Formerly known as Bow House, and close to the workhouse grounds, it was, until acquired by the Guardians, a family residence, and possesses an old-time lawn and garden. It has, of course, been completely renovated, but it still preserves the charms of antiquity, while it has also all the advantages essential to a well-ordered and well-arranged modern establishment. In no sense, however, is it conventional. The object in view was to provide a real home for the nurses, and this has been completely accomplished. The inmates might fitly describe it as their "country house." A working housekeeper has been appointed by the Guardians, and under the superintendence of Mrs. H. Cooke, the matron, the Epsom nurses will find their surroundings much more pleasant than those of most of their profession who are engaged in workhouse infirmary nursing.

### "SAIREY GAMP" IN IRELAND.

That the "Sairey Gamp" tribe is not yet extinct in the sister island is shown by an account in the *Irish Times* of the proceedings at a meeting of the Rathdown Board of Guardians. The report of the House Committee stated that Nurse Kelly, wife of the workhouse clerk, who is in charge of the fever hospital, had five of her children living in that portion of the house, and attending the National School. With one exception the Guardians recognised the need of an alteration in this state of things. Mr. Kennedy thought that "they had more pressing evils to look after," but the chairman of the committee and other Guardians insisted upon Nurse Kelly removing her children. If the nurse had been trained, she would certainly have known better than to keep her children in a fever hospital, where the peril to themselves must be as obvious as the danger to those with whom they come in contact.

### THE QUEEN'S NURSES AT CARDIFF.

It is now nine years since the Cardiff and District Branch of the Queen's Jubilee Institute for Nurses was started. How great the need must have been for the movement may be judged by the fact that in 1891, the first full year, the number of visits made was 15,434, while for the ten months of 1899, up to the end of October, it was 27,296. During the entire period the number was considerably upwards of 200,000. The amount of work done by each nurse must be heavy, for at no time have more than nine been employed. Unfortunately, popular as the nurses are among all classes, the institute only just pays its way, and the demands upon it have become more than the staff can meet. For a wealthy town like Cardiff, which is growing by leaps and bounds, and getting richer as it grows, £450 is a miserably small sum to contribute towards the provision of nurses for the sick and poor.

### THE HEBREW WARDS OF THE LONDON HOSPITAL.

The London Hospital, from its close proximity to the East India Docks and the teeming foreign population of the East-end, is, perhaps, the most cosmopolitan of our great hospitals, and this is especially noticeable in the Hebrew wards, which are exclusively used for Jewish patients. There is little to distinguish Rothschild and Goldsmidt from the other wards of the hospital except the Ten Commandments in Hebrew characters nailed above the lintels of the doors, and the seven-branched candlestick above the fireplace for use at Passover time; yet there is a world of difference between the inmates of the wards and the East-enders who are the usual patients. On Saturday the Rabbi conducts service in the two wards, which is always looked forward to with eagerness, for even the youngest of Hebrew patients are not ignorant of their faith. The Hebrew patients are keenly susceptible to physical suffering and discomfort, as all who possess the artistic temperament are, but they are more grateful, and there is a singular freemasonry of suffering, a brotherhood of patient endurance, which makes the ward a little world of its own, speaking its own language and having its own joys and sorrows. The sympathies and interests of the occupants are wide and far-reaching; patients greet one another whose last meeting may have been within the pale or on the Russian frontier. On Friday evening the Kosher candles are lighted, and one of the patients chants the Yigdol. If any in the women's ward are convalescent one will enter, leaning on the nurse's arm, in order to light the candles—the homelight which has always gleamed in the domestic life of the Jews, and shone brightly even amid persecution and trial. Another characteristic which contradicts preconceived notions of the Jews is their willingness and ability to contribute towards the hospital funds. Even the extremely poor will drop a few coppers in the box with many fervent blessings, thereby setting an example to a good many Gentiles.

### SHORT ITEMS.

A new steam disinfector, by Messrs. Goddard, Massey, and Warner, of Nottingham, having been fixed at Queen Charlotte's Lying-in Hospital, the managers are now desirous of disposing of the gas disinfector.—The American hospital ship "Maine," after being surveyed and passed by a War Office expert, sailed for Cape Town on Saturday.—The Hull Board of Guardians, at the instance of Sir James Reckitt and others, have increased their subscription to the Jubilee District Nursing Association from 10 to 30 guineas.—A nursing association has been formed at Bollington, with Miss Bridger in charge. She was previously at Huddersfield, and was trained at the Princess Alice Hospital, Eastbourne, and the Devon and Exeter Hospital, Exeter.

### The Princess of Wales's Nurses for the War.

The warm personal interest taken by the Princess of Wales in the soldiers at the front was shown by the equipment of the hospital ship, the "Princess of Wales," under the direction of a committee of which the Hon. Sydney Holland was chairman. The Princess is honorary president of the Royal Red Cross Society, and in that capacity she determined to select some of the most highly trained ladies, to be known as the Princess of Wales's Nurses, and to despatch them to the seat of the war. She resolved to send immediately twelve nurses, six of whom were chosen from the staff of the London Hospital on the recommendation of Mr. Holland. The latter left for South Africa on Saturday in the "Dunottar Castle," the same ship taking Lord Roberts and his staff. The remainder will leave early in January.

The Nurses at Marlborough House.

On Thursday and Friday the Princess, accompanied by Princess Victoria, received the whole of the twelve nurses at Marlborough House. Her Royal Highness having shaken hands with, and spoken to each of them individually, affixed a white linen badge embroidered by herself in red with two crosses. The upper cross contains the initials of Her Royal Highness, "A. A." entwined, surmounted by her coronet. This forms the special badge to be worn exclusively by the Princess's own nurses. The lower cross on the badge is

The Badge Worn by the Princess of Wales's Nurses.

the well-known Red Cross. Each nurse received two beautiful shawls and a calendar. We are not at liberty to repeat exactly what the Princess said to the nurses, but she expressed her pleasure at seeing them, and her hope that she would receive them all again on their return from the war. She also addressed some earnest words of encouragement to them, which produced a deep impression, and will never be forgotten by any of them.

How the Nurses were Selected.

The wisdom and judgment of the Princess are exemplified by the fact that amongst her twelve nurses are the most experienced sisters from Guy's, St. Bartholomew's, King's College, and London Hospitals, the Home Hospital in Fitzroy Square, and Netley House; whilst the provincial hospitals are represented by a superintendent of nursing, who has had great surgical experience under the late Mr. Greig Smith, of Bristol, and Mr. Pye Smith, of Sheffield. In fact, the whole of the twelve are nurses of the highest type, with complete knowledge of all modern developments in surgery. One sister was a nurse in the war between Greece and Turkey, and holds the Greek war medal. It is to be hoped that the valuable experience possessed by the Princess of Wales's nurses will be turned to the best possible account, and that their skill will be utilised immediately at the front. We are gratified to be able to state that, without exception, the authorities of the hospitals and institutions from which the nurses have been chosen, have decided to grant them leave of absence during their term of service in South Africa, and to reinstate them on their return.

The Personnel of the Nurses.

Miss Louise Asman was trained at the North-West London Hospital for three years, and from 1895 to the present date she has been staff surgical nurse at Fitzroy House Home Hospital, where she has nursed every kind of surgical and operation case under leading London surgeons.

Miss Becher is "Sister Mellish" at the London Hospital.

Miss Mary Isabella Burdett was trained at Guy's Hospital and Addenbrooke's Hospital, Cambridge. She was matron for a short time at Caldecote House Convalescent Home, Bushey Heath, and from October, 1898, to the present date she has been charge nurse, nursing operation and surgical cases only, at Netley House, 15, Henrietta Street, Cavendish Square, being in charge of cases under most of the leading London hospital surgeons during this period of her career. Miss Burdett is a sister of Sir Henry Burdett, K.C.B.

Miss Amy E. Davidson was trained at Guy's. She has been five years a nurse and two years a surgical sister in charge of the operation theatre. She was a nurse in the Turco-Greek War, sent out as one of the Princess of Wales's nurses and has acted as night nurse in charge of surgical wards. Miss Davidson's father is chaplain to Middlesex Hospital.

Miss Evans is a staff nurse in Mellish ward of the London Hospital.

Miss Greenham is "Sister Mary" in the London Hospital.

Mrs. Kelso Hamilton is a staff nurse in Mellish ward of the London Hospital.

Miss Mary Frances Lightfoot was trained at the Infirmary, Myrtle Street, Liverpool, and King's College Hospital, London. Since 1894 up to the present time she has been Sister to the operation theatre and in charge of surgical wards at King's College Hospital. Miss Lightfoot is a daughter of the late Dr. Lightfoot, Rector of Exeter College, Oxford.

Miss E. McCarthay is "Sister Sophia" at the London Hospital.

Miss McGowan was appointed "Sister Rothschild" at the London Hospital six months ago, having previously been receiving-room sister.

Miss Louisa Barry Peers was trained at the Royal Infirmary, Bristol, where she had exceptional experience in the nursing of surgical cases under eminent surgeons. She was for three months surgical sister at the Accident Hospital Llanelly, North Wales, where she attended all the operations. She is at present superintendent of nurses at the Infirmary, Dudley. The father of Miss Peers was formerly surgeon-major in the 5th Royal Irish Lancers.

Miss Jane Elizabeth Skillman was trained at St. Bartholomew's Hospital, and was subsequently night sister for two years, and since 1884 up to the present time has been sister of Hope Ward. Whilst being a most admirable medical nurse, Miss Skillman's surgical experience has also been most ample.

### To Nurses.

In order to increase and vary the interest in the *Mirror*, we invite contributions from any of our readers in the form of either an article, a paragraph, or information, and will pay a minimum of 5s. for each contribution. All rejected manuscripts are returned in due course, and all payments for manuscripts used are made at the beginning of each quarter, *i.e.*, January 1st, April 1st, July 1st, and October 1st.

### Lectures to Nurses.

### THE MANAGEMENT OF NORMAL LABOUR.

By ROBERT JARDINE, M.D., &c., Physician to the Glasgow Maternity Hospital.

A NORMAL labour may be defined as one in which the vertex presents, and the child is expelled within 24 hours by natural efforts without anything untoward happening. The genital tract is, as a rule, aseptic. Our aim is to keep it so during labour and the puerperium. To this end the patient's external genitals must be kept thoroughly clean. Everything which touches the field of operation must also be aseptic, and the internal examinations must be as few as possible. I have found lysol the best antiseptic for general use. It is non-poisonous, and it makes a slippery solution which obviates the need of using vaseline or oily preparations for the fingers.

**Arrangement of the Bed.**—The bed should be protected by a waterproof sheet, and a clean drawsheet. Among the poor you will often have to use paper instead of a waterproof sheet. Where it can be afforded an obstetric sheet or gamgee tissue should be used in place of an ordinary drawsheet.

**External Examination of the Patient.**—Palpation of the abdomen should always be practised before making a vaginal examination. You can thus learn if the patient is pregnant, if she is in labour, whether the presentation is longitudinal or transverse, and which end of the child—head or breech—is presenting. An expert can, of course, make out much more. If the labour could be conducted without any vaginal examination at all the risk would be much lessened, but this is hardly practicable. The internal examinations, however, should be as few as possible, and must be made with every possible care. To sterilise your hands and forearms wash them very thoroughly with warm water and soap, scrubbing them, and especially the fingernails, with an aseptic nailbrush. They should then be immersed and scrubbed in a warm antiseptic solution. Lysol, a teaspoonful and a half to the pint, does admirably. The patient's external genitals and buttocks should then be thoroughly washed with soap and water and swabbed with the lysol. For swabs use tow, absorbent wool, or a clean rag, but never the ordinary domestic sponge. Again sterilise the hands; then, with the patient in the ordinary left side position, raise the upper labium and pass the fore, or fore and middle finger of the right hand into the vagina, without touching anything else. You thus make out the condition of the vagina, whether or not the patient is in labour, the amount of dilatation of the os, the condition of the membranes, the presentation, the position, and the stage of labour. Continue the examination during a pain, but be careful not to rupture the membranes unless the os is fully dilated. If you find everything right tell the patient so; but if you find anything abnormal do not alarm her. Tell some responsible person and get medical assistance. Never commit yourself to any definite time for the termination of labour.

If the bowels are loaded give an enema, and if the bladder is distended get the woman to pass urine. If she fails, pass a catheter, with aseptic precautions. If the labour is likely to be over soon have plenty of hot and cold water at hand, and air the clean sheets, binder, napkins, baby clothing, &c. See that your ligatures for the cord are ready. The best ligature is made of several plies of stout linen thread. During the first stage you may let the patient move about the room. Give her some light nourishment occasionally, but no alcohol. As soon as the membranes rupture put her to bed, and examine vaginally, first sterilising your hands and swabbing the vulva. The cord may have been swept down by the rush of "waters." If so, an attempt should be made to replace it at once before it gets pressed upon. Place the patient in the knee-elbow position, and try to push the cord above the head. If you cannot do this, or if it does not stay there, send at once for assistance.

During the second stage the suffering increases. Encourage the woman to bear down, and fasten a towel at the foot of the bed for her to cling to. Firm pressure over the sacrum will relieve the pain in her back. If the legs cramp, rub them briskly. When the head begins to distend the perineum place a pillow between the knees or get somebody to support the right thigh. To support the perineum place a pledget of tow over the anus, and your right hand flat over this with the thumb at one side of the vulva and the fingers at the other. Press the whole structure well forward, while with the other hand passed between the thighs you grasp the occiput, and keep the head well flexed as it comes out.

When the head is born, feel if the cord is round the neck. If it is, pull the loop slack enough to pass it over the head, or to allow the shoulders to come through. If it is too short to allow of this, cut it, and deliver quickly, so as to get the end next the baby tied as soon as possible. As the body is being expelled try, by pushing the shoulders well forward, to prevent them tearing the perineum. Follow the fundus down, and get somebody to grasp the uterus while you are attending to the child. The patient herself can sometimes do this. Wash the baby's face and eyes, and as soon as it cries and breathes well tie the cord in two places. Cut it on the palm of your hand, to avoid injuring any part of the child. Roll the child in a warm wrap, and place it in safety while you attend to the mother.

**Conduct of the Third Stage.**—With the patient lying on her back, keep gently kneading the uterus, and when you feel the placenta slip out of it press firmly downward and backward, to expel it from the vagina. The membranes are best removed by grasping them between the finger and thumb, and withdrawing them gently. After a little experience you will know by the feel whether they are coming away complete or tearing. The perineum should always be examined as soon as the child is born. If it is torn halfway or further back you must send for someone to stitch it.

As soon as the placenta is away and hæmorrhage has ceased, remove all soiled things from below the patient, cleanse her thoroughly with lysol solution, and apply an aseptic napkin. If you are not sure of the napkin, wring it out of the lysol before applying. If the uterus is firm you may now apply the binder. When the patient is made comfortable you should turn to the child. Wash its eyes very carefully with clean water, and then bathe it, using oil or vaseline if necessary to remove the vernix caseosa. After it is thoroughly dried examine the stump of the cord. If there is any oozing put on another ligature. The cord is usually folded in a piece of clean linen rag, but an antiseptic dressing would be better. In hot climates it should always be treated antiseptically, to prevent tetanus. Do not immediately dose the child with castor oil. It may be put to the breast after the mother has rested a little. If peevish it may have a little sugar and water to drink. Before leaving your patient give her a cup of tea and change the napkin. See that the discharge is not too free. Count her pulse. If it has risen to about 120 be on the watch for post-partum hæmorrhage, and do not leave her. Observe I have not mentioned ergot. I do not think it should be used as a routine practice. It should be given if the discharge is too free or if the uterus shows a tendency to relax, but never until the placenta is expelled. In our next lecture we shall deal with the care of the patient during the puerperium.

### Christmas Eve at an Ophthalmic Hospital.

By a London Nurse.

It was Christmas Eve in London, and since daybreak the snow had been falling, till, as the afternoon closed in, the whole of the metropolis was covered in a soft coating of white. The big clock from the church which stood close to one of the ophthalmic hospitals struck four. The patients were having tea, and they were all unusually quiet.

Many of them were in bed with bandages across their eyes, and some had been operated on that afternoon, so were feeling the depressing effects of an anæsthetic. But somehow, perhaps because the spirit of Christmas seemed to pervade the air, after a time, even the most woeful among them began to look at matters in a less dismal light, and one old man of seventy-two quavered out "Auld Lang Syne" to an appreciative audience.

In Oxford Ward there was an old woman who for months past had suffered agonies in her left eye with rheumatic iritis. For the last half-hour she had been in great pain; and although old "Granny Brown" (as the nurses called her) could not have told you why, somehow she grew calmer and the pain became easier to bear.

The woman in the next bed, who had a bandage across her eyes, put a lean, brown hand out from beneath the clothes and felt on her locker for a feeding-cup of milk. The hand went tapping all over the locker in nervous haste, and then disappeared under the bedclothes again, while Mrs. Ten resigned herself to another half-hour without sustenance.

Granny Brown had a great aversion to Mrs. Ten because she ate biscuits out of a paper bag in the middle of the night, and was always telling Mrs. Nine that if the nurses allowed her to "eat a snack of something" every half-hour she knew that she would have left the hospital long ago. As Granny Brown lay watching Mrs. Ten, she suddenly thought that it would be kind to offer her enemy a little of her milk, because everyone ought to be charitable on Christmas Eve.

But Mrs. Ten refused politely. She said she knew Mrs. Nine's milk was half barley water, so not so strengthening for a delicate female like herself.

"Mrs. Nine!" exclaimed a woman in the bed opposite Granny Brown's, "I'm going' ter arst Sister if she'll put the piece of miseltoe over your bed, 'cos Lizzie said she sawed a stranger in your tea-cup."

Granny Brown smiled faintly. "Bless your 'eart!" she murmured, "I ain't expectin' nobody. My son Tom is at Sheffield with my daughter-in-law's people, an' Lyddy won't let 'im come with a three-days'-old baby an' all. Then Mrs. Croft on'y said in 'er post-card yesterday that she'd try to come, so I reckon that means that she ain't comin' at all."

"'Ave you only got one son?" asked Mrs. Ten, feigning astonishment. "Why! I've got six; an' I expect if they all turn up to-morrer they won't forget to bring their mother some little trifle—bless 'em!"

Just then Sister Agatha came in to put a boracic fomentation on Granny Brown's eye.

"How is the pain?" she asked, as she replaced the bandage.

"It's bin crool bad, dear, but I'm a bit easier now. I just wanted to arst you if Mrs. Croft didn't say in the letter-card you read me yesterday that she'd try to come, not she was comin'?"

Sister Agatha turned very white, and she drew her lips tightly together. She had deceived Granny Brown about the letter-card which had come just at this time yesterday, and, although she had judged she was doing it for the best, still she had wished ever since that she had not been obliged to resort to strategy. It happened in this way: Just after the patients had had tea a letter-card came for Mrs. Nine, and Sister Agatha took it to her at once. But Mrs. Nine said she was afraid of using her "good eye" to read it.

"I expect it's from my son Tom or Maria Croft," she said quietly. "Prap's you'd read it, Sister, if you can spare a minute."

So Sister Agatha had torn off the perforated edge, and it took her only half a minute to hastily take in the contents. It ran thus:—

"Dear Eliza,—Poor Tom died this mornin'. 'E were rather queer on Sunday woodnet touch is food or nothin i did my best for im but it was no good cos ed got pew moanyer i expect it will be a sad Chrismis for you. I will try to cum and see you on Chrismis Day and giv you the news.—Yours trewly, Maria Croft."

Sister Agatha could never forget her feelings as she stood with the letter-card in her hand, pretending (in order to gain time) that she could not read Maria Croft's handwriting.

"Let me try an' read it," Granny had persisted, so Sister Agatha had done what was best. When she read it out to Mrs. Nine it ran thus:—

"Dear Eliza,—I will try and see you to-morrow or Christmas Day. I hope you are not worrying yourself.—Yours affectionately, Maria Croft."

"Thank you, dear," said Granny, "you can burn it now if that's all she says. She do seem to get on with 'er writin'—quite like an eddicated person, aint it? But there—Maria allus was a pushin' woman. She tikes in washin', you know. I've seed Maria make a bar of soap go twice as far as anyone else; that's brains!"

But Sister Agatha had not even smiled, or stopped to chat as she sometimes did. She was too busy turning over in her mind when and how Mrs. Nine should be told of her loss. At any rate, she had spared Granny Brown the shock of reading the letter-card herself. So now, when Mrs. Nine referred to the letter-card, Sister felt the same agitation she had experienced the day before. "Mrs. Croft said she would try to come," she said quietly as she moved away.

* * * * *

For the last week there had been great preparations for Christmas going on in the Eye Hospital. There were numerous brown paper parcels for the patients; not quite as many as Matron would have liked to have received, because so many people are either ignorant, or strangely forgetful that in an eye hospital most of the patients are condemned to lie hour after hour with bandaged eyes, or even if able to be out of bed, and with only one eye affected, are not allowed to read or work. It is this that makes Christmas Day so sad for those who cannot use their eyes, for they are unable to see any of the festivities or properly appreciate their presents.

In the smallest crib of the hospital lay the youngest patient—a little boy of three years old, who had been admitted only the day before with a burnt eyelid.

He whimpered a little in his sleep, and drew in his breath with a shivering sob. "Want mumma an' the knife wif the corkeely kroo," was the scarcely audible demand. And just then Sister stooped down and kissed him, and he smiled in his sleep because he felt her there.

The church clock had just struck six, and Mrs. Nine was preparing herself for a little doze, when the ward door opened and in walked Tom Brown.

"Haloo! old mother! ain't this a surprise? I got 'em to let me in 'ere. Give us a kiss. Lyddy would 'ave me come; baby's a fair beauty—weighs seven pounds—"

"Oh! Tom! exclaimed his mother, "You've fair took my breath away comin' in so sudden, an' you the last person I expected to see. Why! there's Mrs. Croft—come in Maria, do!"

Mrs. Croft had been waiting outside till the first burst of excitement was over, and Sister Agatha had been peeping through a chink in the door, with such a merry twinkle in her eyes, that Maria Croft said afterwards she was "a fair treat to look at."

"Did you get my card, Eliza?" exclaimed Mrs. Croft. "Weren't it sad about Tom? I'm goin' to 'ave 'im stuffed, 'cos 'e was such a pet of yours! 'E was an artful cat (them Thomas ones allays is), but 'e 'ad 'is good points."

"Why, I never noo 'e was dead!" gasped Mrs. Nine.

"Lor' bless my soul! I put it in the card. 'E went off with the pew moanyar, all caught by sittin' on a water-barrel all night, pore beast."

And then Sister Agatha came in and explained everything amid much laughter. Granny Brown said she had never laughed so "'earty" about anything before.

"So now I suppose you'll 'ave your eye took out an' a glass one put in?" remarked Mrs. Croft, as she laid out a few packages on the bed, while Tom cut the strings and displayed their contents.

"You get Tom stuffed first, and then I'll see about my glass eye," laughed Mrs. Nine.

And as the visitors walked away, they felt what they would never have put into words, their gratitude that "Granny" was doing so well.

### Novelties for Nurses.

### AN IDEAL SHOE FOR NURSES.

THERE is probably no period of her toilet so much grudged by a nurse as the time she is obliged to spend in fastening her shoes. It always happens that just at the critical moment either the lace breaks, or if buttoned a button comes off, and her progress is delayed in an irritating manner. To remedy this state of affairs the manager of Messrs. Gooch's Stores in the Brompton Road (67 to 77) has recently produced from the design of a trained nurse a shoe which promises to eclipse all others in the market for elegance of design combined with the advantage of slipping on and off with the greatest ease and without any trouble of fastening. In appearance the shoe looks like an ordinary well-made laced-up walking shoe, but on either side of the laced-up front, which is purely ornamental, there are cunning little gussets of stout elastic which admit of the shoe being slipped on and off without any difficulty whatever. Thus while a nurse is removing her cloak and bonnet she can at the same time be divesting herself of her shoes, and by the time she has adjusted her cap she can also have assumed another pair (we always recommend two pairs in use at the same time) if she desires to change. Though primarily intended for ward wear, these shoes can be used with equal comfort out of doors. In addition to the speciality we have just described, the shoe is provided with all the advantages of Messrs. Gooch's "Samaritan" ward shoe, of which we gave a description in a recent issue. The sensible square military heel, rendered noiseless by the insertion of a small wedge of indiarubber, the instep arch supporter, are all part of the features of this ideally-perfect shoe for nurses, which bears the name, by Her Royal Highness's gracious permission, of "the Princess Christian Ward Shoe." The shoe is made in the softest glacé kid, and is delightfully soft and yielding to the foot. Though intended for nurses, there are few busy women that will not hail its production with delight, as for convenience as well as comfort it has never been surpassed. The price is wonderfully moderate at 12s. 9d. a pair, but it is hoped thereby that they may be brought within the reach of all.

### Appointments.

DUFFERIN VICTORIA ZENANA HOSPITAL, CALCUTTA.—Miss Ada M. Rawlings has been appointed Lady Superintendent. She was trained at Edinburgh Royal Infirmary. She has since been Sister at the Marylebone Infirmary, at the National Hospital for the Paralysed, London, and also at the General Plague Hospital, Poona, the Arthur Road Hospital, and Parish Plague Hospital, Bombay.

ALLT-YR-YN FEVER HOSPITAL, NEWPORT, MON.—Miss B. L. Greenland has been appointed matron. She was trained at Worcester General Infirmary, and has since, for three and a-half years, been sister at Allt-yr-yn Fever Hospital.

GREAT NORTHERN CENTRAL HOSPITAL, LONDON.—Miss Alice Humphrey has been appointed Assistant Matron. She was trained, and has been staff nurse four years and ward sister seven years, at the same institution.

### Christmas in the Hospitals.

It would probably startle a good many people to be told that the place to spend a happy Christmas in is the ward of a large hospital. The popular idea still is that the "poor creatures" in the hospitals deserve particular sympathy at the most festive season of the year. So they do, on account of their sufferings; but in all other respects they are really much more to be envied than to be pitied. This is, of course, because no section of the community enlists so many clever heads, so many deft hands, so many kind hearts, on its behalf as the patients in our hospitals. At all times the wards of modern hospitals are made as cheerful as circumstances will permit, but on Christmas Day they are often like fairyland. Nor is it merely that the decorations are artistic and original, but on every side there are practical evidences of the interest which is taken in the inmates—the desire to cause them to feel that they are at home in the truest sense of the word. The central idea of all who contribute to the sum of enjoyment is to impress every man, woman, or child in the building with the conviction that they are joining in a family gathering. Those who had the privilege of visiting any of the great institutions in London, such as Guy's or the London, on Monday, will readily understand what we mean. The moment you entered the doors you recognised the force of the contention that the hospital is a potent humanising influence. An irresistible air of cheerfulness dominated everything and everybody. You grasped the situation—that one set of persons were doing their utmost to afford pleasure, while the other set were doing their best to show their appreciation of the efforts on their behalf. It is largely because the work of ministration is so unaffectedly and heartily welcomed by those who are ministered unto, that each succeeding Christmas seems brighter and happier than the last. The mode of entertaining the patients vary. At the London a prominent feature at Christmas is carol singing by the nurses, whose sweet voices literally make music in the wards during the early hours of the morning. Flowers and fairy lights help to give the wards a charming appearance; but the remarkable excellence of the decorations at Guy's—which have been a study for years—goes far to kill the decorations at other hospitals. The ingenuity manifested in the choice of the designs is not less noteworthy than the care obviously shown in matters of the smallest detail. This spirit of thoroughness is discernible throughout. While everything is done spontaneously, the single object of affording the maximum of gratification, with no idea of saving trouble, is always kept steadily in view. The band of merry medical students, who, capitally got up as a minstrel troupe, perambulated the wards of Guy's for upwards of six hours, followed by groups of delighted spectators, cannot be too warmly praised for their attention to trifles. They frequently displayed talent of a high order, but the dresses were as elaborate, and the jokes as neatly turned as if they had been a company of trained professionals depending upon their merits for their remuneration. While some of them sang for the benefit of all, others indulged in by-play for the diversion of the children, or for patients who were supposed to have a preference for the fun. It might have been imagined that the noise of the entertainment would be trying to some, but the habitués of hospitals are accustomed to noise wherever they are, and the suggestion to a patient who was clearly in great pain that the entertainers disturbed him provoked quite a pathetic protest.

But it is not in a particular way, so much as in all ways, that matrons, sisters, and nurses, medical superintendents, and students, even the visitors themselves, seem to derive happiness in making others happy. The Christmas trees, the flowers, the pretty presents, the good fare, the kindly greetings, the thoughtful inquiries, the perpetual indications of loving service which characterise the staff of a hospital at Christmas—and of which examples will be given after the close of the festivities in these pages—may seem of small moment to the cynic, but their effect upon the body politic is none the less immense; for every happy Christmas spent in hospital must tend to broaden the great stream of human gratitude, which in its turn unlocks the fountain of human love.

### Presentations.

SKIPTON HOSPITAL.—Miss Alice Riley, who has just been appointed Matron of Skipton Hospital, has been presented by friends and patients in Skipton with a solid silver set of brushes, a mirror, comb, pin tray, silver-mounted purse, and a Prayer Book. Miss Chevallier, on her appointment as Nurse at the hospital, has also been presented with a travelling clock and other articles. Nurse Chevallier worked as district nurse for two years and a half.

TRAINED NURSES' INSTITUTE, WEYMOUTH.—Christmas at this institute was a very happy one. The nurses, as usual, were invited to dinner or supper by the superintendent, Miss Dibb, and the latter was presented by the nurses with a very handsome Crown Derby salad bowl and servers, mounted in silver. Miss Cornwall, the matron, also received a handsome marble clock with gilt figures.

ROYAL INFIRMARY, EDINBURGH.—On Friday last Miss Lawson was made the recipient of a solid silver tea service and numerous other presents by the lady students and residents of the ward which she is in charge of in the Royal Infirmary, Edinburgh. Miss Lawson is leaving Edinburgh to fill the post of lady superintendent of the Stanley Hospital, Liverpool.

CARDIFF INFIRMARY.—On Christmas morning the sisters and nurses of the Cardiff Infirmary presented their matron (Miss Wilson) with a complete writing table set, comprising a silver inkstand, pen-holder, pencil-holder, paper knife, and stamp box, as a mark of their affection.

### Minor Appointments.

ROYAL UNITED HOSPITAL, BATH.—Miss S. Wales has been appointed Night Sister. She was trained at Bristol General Hospital. Subsequently she was for two years on the private staff of the Bristol General Hospital, and has since been in charge of the female floor and theatre, Dorset County Hospital.

GUEST HOSPITAL, DUDLEY.—Miss Fraser has been appointed Night Superintendent. She was trained at the Metropolitan Fever Hospital, Hampstead, and the General Hospital, Wolverhampton. She has since taken sister's holiday duty, and had charge of the fever block at the latter institution.

HORTEN HOSPITAL, BANBURY.—Miss Nancy R. Mackintosh has been appointed Charge Nurse of medical and surgical wards and theatre. She was trained at the Royal Infirmary, Perth, and has since been nurse at St. Luke's Hospital for Diseases of Women, Edinburgh.

ST. SAVIOUR'S INFIRMARY, EAST DULWICH GROVE.—Miss Margaret Elliott has been appointed Sister. She was trained at the Western Infirmary, Glasgow, and was subsequently head nurse at the Lady Hozier Convalescent Home, Lanark.

### Where to Go.

THE HAMPSTEAD CONSERVATOIRE, SWISS COTTAGE.—Monday, January 8th, half-past eight p.m.: Reading of "Hamlet," by Mr. Forbes Robertson, in aid of the Hampstead Hospital.

THE LIVINGSTONE EXHIBITION, St. Martin's Town Hall, Charing Cross, January 1st to 5th.—Nurses in uniform admitted at half price.

### Echoes from the Outside World.

### AN OPEN LETTER TO A HOSPITAL NURSE.

It seems hard lines that the Duke of Connaught's desire to go to the front should be thwarted. At the outset he was anxious to be employed, and would not accept non-compliance with his request as a final refusal. Having met with a similar answer to a second application to the authorities, he asked Lord Roberts if he might be attached to his staff "in any capacity in which he could be useful," quite independent of his military rank. In this instance he appeared to be more successful, for the gallant Field-Marshal willingly consented, but again a higher power intervened and barred the way. It is not difficult to understand the reasons of the authorities, while one sympathises with the Duke. The Queen is the last person to try to prevent him from going, but it may, none the less, be considered important not to add to the anxieties which she already suffers. Perhaps, too, the presence of one of her sons at the front, exposed to danger, would needlessly increase the sense of obligation felt by the officer in command; while, finally, if he is wanted at home—in London or in Dublin—there is a solid consolation for his natural disappointment.

From the front there is no news, except that Ladysmith, Kimberley, and Mafeking still hold out, the latter getting more and more hard pressed. All the British commanders seem to be waiting fresh developments before striking again, General Gatacre alone having made a decisive move, by occupying Dordrecht without any casualties. Meanwhile, Lord Roberts left Waterloo on Saturday, looking, in spite of his son's death, bright and fit as he responded to the Prince of Wales's affectionate "Good-bye, Bobs." The Volunteer movement grows mightily as it rolls along. There is already £73,000 at the Mansion House to equip the City of London Imperial Volunteers, and hundreds more men than can possibly be accepted. Colonel Mackinnon will command. Westminster also hopes to supply £20,000 to equip a thousand volunteers for service at home or abroad, so the capital of the Empire is doing nobly.

You have probably before this discussed amongst yourselves the advisability of a day of national "humiliation." The idea has not on the whole been received with the degree of favour which those who put it forth anticipated, and the probability is that the proposal will fall through. Decidedly, the word selected as descriptive of a day set apart for the acknowledgment of any faults of arrogance, of which as a nation we may have been guilty, and for beseeching the Almighty to give us His aid and protection in the campaign, is singularly ill-chosen. Because, during a war of less than three months, we have failed to prevail over a foe which has been making hostile preparations against us for some years, there is no cause for "humiliation." That our religious belief, if it is a real one, should lead us to pray incessantly for the help of that Lord, who was once declared by His prophetess to be a "Man of War" no Christian will deny, and to this even those who have lost their early faith have no objections to raise, but one day of "humiliation," followed by no special perseverance in prayer, could have no lasting results. The misfortunes we have met with assuredly have been for our ultimate good, and we shall reap the fruit of them in due season; but I do not think that they have in any sense humiliated us. Rather, whilst overshadowing us with the clouds of sorrow, they have revealed to us too the silver lining —namely, the thought that the nation can still suffer and be strong, and that the enthusiasm of our men and the unselfishness of our women have not died out as some imagined, but are as strong now as they were in the terrible days of the Crimea, or in the dark weeks of the Indian Mutiny.

The brightness of the Christmas season has not been shadowed only by the war in South Africa. On Saturday came the tidings of a terrible avalanche at an Italian winter resort involving the loss of a number of lives, including those of two English ladies who were staying at Amalfi. Just imagine the terror which the sudden fall of a rock of enormous dimensions, carrying with it a huge hotel, a monastery, and other buildings, must have caused! Moreover, the work of destruction did not end here, for several waiting vessels anchored near the spot were crushed to the bottom of the sea by the wreckage. The amazing thing really is that so many persons escaped. The English ladies, one of whom was a daughter of Mr. Weir, M.P., would, it seems, have been among these if they had not insisted upon getting a portfolio containing 6,000 francs before quitting the Cappuccini Hotel. Poor things! Even if the 6,000 francs had been all the money that they had in the world, it surely was not worth while to risk their lives.

Then, on Christmas Eve, we were all startled at the breakfast table by the news of three disastrous railway accidents. That on the Brighton line came home to us especially, because the trains in collision were the Pullman and the boat express from Newhaven, which people near suburban stations are accustomed to see rush through out and home at terrific speed. The management of the local traffic on the Brighton lines has lately been rather worse than usual, and the collision at Wivelsfield, following one at Bermondsey a week earlier, suggests general carelessness on the part of the authorities. Of course, the blame is put on to the fog; but surely it is the business of railway managers to protect their passengers most securely in the time when the elements of risk are obviously greatest. From a passenger in the Newhaven boat train, a neighbour of ours, I learn that the effects of the collision would have been far more disastrous even than they were if the train had been standing still instead of moving. He was in one of the front carriages, and was only slightly hurt in the arm, not sufficiently to prevent him from assisting in the rescue of a poor man from Croydon, who had to be sawed out of an overturned carriage. Everybody who could, he says, worked hard to help the injured, but the scenes were most distressing. Some who escaped direct injury from the collision itself were hurt by heavy luggage, thoughtlessly placed in the racks for wraps, being hurled against them. It is a pity that such plain disregard of the company's regulations cannot be punished.

Have you ever noticed how often notable people die at the end of the year? In quick succession there followed last week the death of General Sir Gerald Graham, Sir Richard Thorne Thorne, that dear old bookseller Mr. Bernard Quaritch, and the Duke of Westminster. I remember an incident characteristic of the Duke. Just after his marriage to his second wife he was staying at Clovelly. The people in the picturesque little village had not the least idea who he was, and one of the natives, who afterwards discovered his identity, told me how he would talk to the fishermen in the most friendly manner. "He was a nice, pleasant-spoken gentleman," said the owner of one of the best boats on the beach, "but he was dressed so like other persons and joked with us so that I never dreamt he was a duke." I don't know how the good man expected a duke to dress—perhaps he alluded to the bowler hat, by no means in its first youth—but I was much interested in watching the newly-married couple stroll about the district. The devotion of the duke to his fair young wife was very apparent, and his kind face was often lighted up with pleasure as he pointed out to her some of the many beauties of that sweet part of Devonshire.

### A Nurse's First Christmas in Hospital.

"What Thou hast given me, Lord, here I bring Thee,

Odour and light, and the magic of gold;

Feet which must follow Thee, lips which must sing Thee,

Limbs which must ache for Thee, ere they grow old."

—*Kingsley.*

Nurse Philippa, or Nurse Phil, as her friends called her, had been just three months a probationer when she spent her first Christmas in hospital. No convict sentenced to three months' hard labour had ever carried out his sentence with greater exactness than had Nurse Phil in those three fleeting months from September 25th to December 25th. Yet, unlike the convict, she had found nothing irksome in her labours; but on the contrary, she had scrubbed lockers with zest, polished tables with zeal, and made beds with enthusiasm. Now, what would her Christmas be like?

She went to bed on Christmas Eve feeling a little sentimental. She intended to think about a great many things—Christmases of the past, home, the vanished scenes of youth. But Nature put an abrupt, if kindly, end to these reflections. The day had been an unusually hard one (not to say a "wild rush"), and just as her head touched the pillow she suddenly fell asleep.

### The Calling Bell.

She had been asleep about ten minutes, she supposed, when some noise awoke her. She was naturally annoyed at being disturbed so soon; but never mind, she would be asleep again in less than a minute; but what, what did she hear? Christmas chimes? Waits? Carols? No, no; the clang, clang, clang of the calling bell left no room whatever for illusions. She had slept all night! It was now a quarter to six a.m., time to be up again and doing. Battling with a weariness, which seemed at first invincible, she tumbled up; no need to strike a light, for there was the waning moon making wry faces at her through the frosty window-pane.

Then all at once a sense of exhilaration seized her, as she realised that this was Christmas Day at last. For weeks the nurses had planned and toiled to make it a day of rejoicing and of relief from the monotony of their lives to the sufferers under their care. And now the time had come, what matter for aching limbs and stiff, cold fingers?

### Carol Singing.

At a quarter-past six the nurses, all in spotless uniform, were assembled in the vestibule. Matron was there, too, her bright red shawl lending a speck of warm colour to the scene. Most of the day nurses, between fifty and sixty, were there, and even a few of the doctors, who had managed to turn out in time. They were going the round of the hospital, carol singing to the various wards. At a given signal from the conductor they struck up the "First Noël." They began at the surgical side of the building, at the foot of the great staircase. From where Nurse Phil stood she could see a large oil painting of the Nativity, wreathed round with evergreens, which made her feel all nice and Christmasy.

"Noël, Noël, Noël, Noël,
Born is the King of Israel."

Many of the patients came out to listen, some in wheeled chairs, some on their crutches, and one or two children escaped and peeped between the banisters. Faces which many of the nurses had grown to love alight, with pleasure at the welcome songs of Christmas. Leading from the surgical half of the building to that devoted to the medical wards there runs a long stone balcony, as everyone knows who has been there, and across this fluttered the procession of nurses, singing with all their might, beneath the frosty moonlight as it struggled with the dawn. The air was sweet and "caller"; there were red streaks in the east, shot with gold, and the few remaining stars shone faintly here and there, like jets of light which no one had remembered to turn out.

From the medical wards a good many of the patients, both men and women, were well enough to come out into the corridors and listen. And many a wrinkled, careworn face softened, and eyes, hard with the fierce battle against fate, grew dim with refreshing tears.

"And ye, beneath life's crushing load,
Whose forms are bending low,
Who toil along the climbing way
With painful steps and slow,
Look now! for glad and golden hours
Come swiftly on the wing;
O, rest beside the weary road,
And hear the angels sing!"

### Breakfasts and Temperatures.

By half-past seven the day nurses were all on duty, just as if nothing had happened, for breakfasts must be given and temperatures taken even on Christmas Day. Nurse Philippa was working then in one of the surgical wards, a very bright one containing 20 beds, 10 on each side of the ward. It was well lighted and lofty, and at the end, on either side of the fireplace, glass doors opened out on to a balcony, where the patients could sit on sunny days. Now the sun was streaming gaily in, for the morning was well advanced, and everything was neat and spotless, while the evergreens and flowers lent to the whole a very festive appearance. The general tone of the ward was cheery, and to-day the patients were a little inclined to be boisterous.

"I say, Davy," called out Moore, the Yorkshire engine-driver, who had never lost his spirits, whatever else he may have lost, along with his leg, in the great accident to the Scotch express a few weeks previously. "I say, Daävey, do you know what maäkes it such a foine day?"

"Na, I dinna ken," replies Davy, who responds to all Moore's sallies in the same way; for he does not understand the Yorkshireman's language, and cannot learn it.

"Whoy," says Moore, in his best drawl, "'tis t' sun's caught t' reflection from our noorse's faäce."

Nurse Phil, who was touching up some flowers, responded to this original compliment by lightly tossing one of the chrysanthemum blooms at Moore's curly head. Then she tried to look as if she had not done so, for here was the Professor entering the ward, with staff nurse and surgeon following in his wake. She flew to fetch the dressing tray, and Moore put the chrysanthemum under his pillow.

### Christmas Dinner.

Christmas dinner in hospital is always more or less of a disappointment, owing to the fact that the inventor of the Christmas dinner (whoever he may have been) did not take the digestive organs of the human body sufficiently into consideration to make the thing a universal success; but it was got over somehow, and then came the blessed, comforting pipes, which make up for so many discrepancies, and Nurse Phil went down to her own dinner.

The nurses' dinner-table had been considered, and was gay with fruit and flowers. At the commencement of the meal the matron had said a few kind, encouraging words to her nurses, and then read out a message of Christmas congratulation from Miss Florence Nightingale to all the nurses there assembled. Nurse Philippa felt a thrill of enthusiasm run through her at thus being actually spoken to, as it were, by the heroine of her girlhood. She thoroughly enjoyed her Christmas dinner, and one of the things that she liked most about it was that it was soon over, and arm in arm with her favourite nurse friend she was flying upstairs to her happy work again.

THE PATIENTS' VISITORS.

The afternoon was taken up by the patients' visitors, and Phil was left in charge of the ward for two hours. Now there came a little time for rest; so, with a book in her hand, she seated herself on the deep window-seat next to Moore's bed, and became the unobserved observer of many quaint or touching little incidents. Davy, for instance, opposite, who had been in the dumps all day, poor lad, cheered up amazingly at the sight of a fat aunt dressed in aggressive mourning, who smelt horribly of peppermint. Still she was a link with home, and such links, however unattractive in themselves, are not to be despised on Christmas Day.

"I warrant there 'll be none o' them coming to see me, Noorse," said Moore, as he caught her eye looking up from her book. "Never mind, Moore," said Nurse Phil, cheerfully; "you can pretend that I am your visitor. There's nobody coming to see me either, so we are both in the same boat." "And there ain't noäbody that I would loike better to be in saäme boät with, except, mebbe—but, theer, what's the use of thinking o' sheä; and she 'ud be put about to see me lying here with my leg under this 'ere blessèd birdcage, and only haäf a leg at that. Heigh dear me! Will ye taäk an apple, Nurse?" "Not just now, thank you, Moore; but tell me, was it your wife you were speaking of?"

"Woife, that was to be. Ay, ay; but it's a question if she'll take a man with only one leg to go dot-and-go-one with, as the saying is." "Oh, I expect she will," said the nurse, warmly. "Well, she mout, and then again she moutn't. By golly!" he cries, suddenly raising himself on his elbow and staring a little wildly at the door.

"What is it, Moore?" "Whoy! if it ain't she hersen'. Well, if I ain't blest!" A fair-haired, rosy-cheeked young woman, blushing to find herself in such strange company, was entering the ward, looking nervously at the rows of beds. Phil went to her and led her to Moore's bedside, and turned away, thinking that there was very little doubt but that the engine-driver's love would be true to him, in spite of the trifling loss of a limb or so.

Nurse Phil soon found herself listening to the odds and ends of conversation which met her ear out of the general buzz. McPherson, the Pott's fracture man, was discussing the merits of the different doctors he had been under with his friend, a clean-shaven old man, who had probably been a patient himself in his time. "Ay, ay," McPherson was saying, "he's a vera quiet man is Jamieson"—hum, hum, hum, buzz, buzz—"and I'll tell ye anither thing aboot Jamieson, he's no afraid to handle a sair" (sore). "Noo, there's plenty o' these young students that's like to tak' a knife and fork ti'lt, but that's no Jamieson, he's no afraid to grip it." "Ay, ay," responded his friend, "he's a cannie mon. There's a hantle a good doctors in an' oot, but what I canna thole is they doctor-esses."

"MY FEYTHER."

At this moment Nurse Phil's attention was called to a visitor who was entering the ward in an advanced state of intoxication. "I suppose he ought not to come in," was her first thought, "but as the porter let him in I don't quite see how I am to turn him back." It was the father of little Andrew, the hip-joint boy, close to whose bed she was standing.

"It's my feyther, it's my feyther," cried the little fellow, his cheeks flushing with delight; "but I doot he's fou. Get him a chair, nurse, and he'll be quiet with me," he added, with evident anxiety for the family reputation. Phil brought a chair and planted the somewhat shaky visitor upon it firmly, and, in spite of strenuous endeavours on his part to shake hands with her, managed to avoid that ceremony without offending him. Presently she saw a slatternly-looking woman approaching, whom she recognised as Andrew's mother. She knew that she and the father were not on speaking terms, for Andrew had told her of the quarrel, and she watched her approach with some anxiety. Little Andrew was clearly overjoyed at the sight of this old rag of a mother, who bent over and kissed him very tenderly. She took no notice of her husband, except to give him an off-hand nod. But when the bell rang for the visitors to go this strange pair departed lovingly arm in arm.

AN ENTERTAINMENT IN THE WARD.

In the evening, after tea, there was a little entertainment in the ward. Some of the students came in and acted a little play, which went off brightly and well. Then old Jock Sutherland, an ex-patient, stood up and sang in his quavery old voice "The Bonnie, Bonnie Banks o' Loch Lomond," and made everybody cry. He was quickly followed by one of the doctors, who sat down at the piano and sang "There were two little owls sitting in a barn," &c., whereupon everybody cheered up and smiled again, forgetful of the broken-hearted bard of Loch Lomond. After that one of the students told "How Bill Adams Won the Battle of Waterloo," which, though far from being new or original, brought down the house.

All too soon the evening slipped away, and then came "Auld Lang Syne." The night nurses had come on by this time, and everybody sang together, fresh young voices and cracked old ones—a pathetic mingling almost too much for excited nerves to bear. Immediately after the lights were turned down, and Christmas Day was over.

### The Nurses' Bookshelf.

[We invite Correspondence, Criticism, Enquiries, and Notes on Books likely to interest Women and Nurses. Address, Editor, THE HOSPITAL, (Nurses' Book World), 28 & 29, Southampton Street, Strand, London, W.C.]

THE OPEN-AIR TREATMENT OF PHTHISIS. By W. BEZLY THORNE, M.D., M.R.C.P. (J. and A. Churchill. Small 8vo. Pp. 67. Price 9d.)

THIS little book consists of two lectures delivered before the Royal British Nurses' Association, and, as the substance of the lectures is very well put and the subject much in evidence at present, we are sure that Dr. Thorne has acted wisely in presenting this contribution to the open-air treatment of consumption in book form. The first lecture opens with the following sentence: "The degree in which the treatment of phthisis has, up to the present time, been committed to the hands of trained nurses is small in proportion to the prevalence of the disease." This is true, and curious as it is true; but it may be hoped that these lectures by Dr. Thorne and Dr. Daw's admirable little work on "The Nursing of Consumptives" will show the trained nurse what a large field she has neglected and what an amount of good she is still destined to accomplish. An extremely clear and interesting account is given of this hospital at Falkenstein, the treatment carried out, and the usual routine of the life therein. The picture recalls every incident connected with a visit we lately paid to it, and makes the whole subject live again for us. What strikes a stranger more than anything else is the fact that the prevalence of cold, wintry weather makes practically no difference in the treatment, and that the recoveries are almost as numerous at one time of the year as another. During winter and summer upwards of 90 per cent. of the patients are out-of-doors during 14 hours of each day, and the percentage of those confined to the house is a mere trifle higher in winter than it is in summer. The second lecture deals with the nurse's part in the prevention of tuberculosis, and Dr. Thorne clearly enough foresees the difficulties in store for the nurse in converting the friends of some of her patients to the new gospel. "Such friends," he says, "constitute one of the patient's greatest dangers, and, difficult as it may be, the nurse can have no more important and honourable task than that of protecting her charge from such ill-advised and pernicious influences."

### The Princess of Wales's War Fund.

We publish to-day the second result of the announcement that we have consented to receive subscriptions from nurses to the Princess of Wales's War Fund, which is even more encouraging than the first. All subscriptions should be addressed to the Manager, Office of The Hospital, 28 and 29, Southampton Street, Strand, London, W.C., and name and address should always be given.

Amount acknowledged last week, £45 6s. 6d.

| | s. | d. |
|---|---|---|
| A. E. Demel ... ... | 2 | 0 |
| S. A. Hancox, P.F. ... | 2 | 0 |
| Martha Bailey, P.F. ... | 2 | 0 |
| Matilda Hill, P.F. ... | 2 | 0 |
| Nurse Boraston, P.F. | 2 | 0 |
| M. H. Dore, P.F. ... | 2 | 0 |
| Isabella Grant, P.F. ... | 2 | 0 |
| E. A. Ridge, P. F. ... | 2 | 0 |
| Nurse Bartlett and Nurse Proctor ... | 2 | 0 |
| T. R. Culliman, P.F... | 2 | 0 |
| M. E. Jones, P.F. ... | 2 | 0 |
| H. Warton, P.F. ... | 2 | 0 |
| Eva F. Finlay, P.F. ... | 1 | 6 |
| M. Hawkins, P.F. ... | 1 | 6 |
| Nurse Ridd, P.F. ... | 1 | 6 |
| A. Howick, P.F. ... | 1 | 6 |
| A. C. Benstree ... | 1 | 6 |
| A. E. Lyne, P.F. ... | 1 | 0 |
| M. R. Hesking, P.F.... | 1 | 0 |
| Blanche A. George ... | 1 | 0 |
| E. Ray, P.F. ... ... | 1 | 0 |
| Ada Northcote, P.F.... | 1 | 0 |
| J. J. Forster, P.F. ... | 1 | 0 |
| E. S. MacGregor, P.F. | 1 | 0 |
| E. Jackson, P.F. ... | 1 | 0 |
| E. M. Goodeve, P.F. | 1 | 0 |
| Elizabeth Forsyth ... | 1 | 0 |
| C. Waterman, P.F. ... | 1 | 0 |
| E. Walker, P.F. ... | 1 | 0 |
| E. H. Harvey, P.F. ... | 1 | 0 |
| A. L. Spreat, P.F. ... | 1 | 0 |
| Nurse MacAllen, P.F. | 1 | 0 |
| Nurse Manby, P.F. ... | 1 | 0 |
| Nurse Jarman, P.F. ... | 1 | 0 |
| Nurse Flint, P.F. ... | 1 | 0 |
| Martha Wiseman, P.F. | 1 | 0 |
| G. M. Sawdon, P.F. | 1 | 0 |
| Mary Cave, P.F, ... | 1 | 0 |
| F. L. Chapman, P.F. | 1 | 0 |
| Miss K. Kent, P.F. ... | 1 | 0 |
| Ellen Snell ... ... | 1 | 0 |
| A. L. V. Balbirniem, P.F. ... ... ... | 1 | 0 |
| Alice Murton, P.F. ... | 1 | 0 |
| Amelia Rolfe, P.F. ... | 1 | 0 |
| M. A. Burdett, P.F. ... | 1 | 0 |
| Louisa Mason, P.F. ... | 1 | 0 |
| M. L. Williams, P.F. | 1 | 0 |
| Emma Still, P.F. ... | 1 | 0 |
| M. Williamson, P.F. ... | 1 | 0 |
| B. E. Shaw, P.F. ... | 1 | 0 |
| L. J. Farrall, P.F. ... | 1 | 0 |
| Mary Bryne, P.F. ... | 1 | 0 |
| Hannah Byland, P.F... | 1 | 0 |
| Nurse Clukey, P.F. ... | 1 | 0 |
| F. A. Albrow, P.F. ... | 1 | 0 |
| C. M. Dowsett, P.F.... | 1 | 0 |
| C. R. Gooding, P.F. ... | 1 | 0 |
| E. F. Pratt, P.F. ... | 1 | 0 |
| Nurse K., P.F. ... | 1 | 0 |
| Mary Fraser, P.F. ... | 1 | 0 |
| Naomi E. Ferrott, P.F. | 1 | 0 |
| Mrs. C. Small, P.F. ... | 1 | 0 |
| G. Hall, P.F. ... ... | 1 | 0 |
| M. Taylor, P.F. ... | 1 | 0 |
| Annie M. Inksore, P.F. | 1 | 0 |
| Nurse Robb, P.F. ... | 1 | 0 |

| | s. | d. |
|---|---|---|
| H. Walker, P.F. ... | 1 | 0 |
| Emily Syles, P.F. ... | 1 | 0 |
| G. Sapcote, P.F. ... | 1 | 0 |
| M. A. Ball, P.F. ... | 1 | 0 |
| Nurse F. H., P.F. ... | 1 | 0 |
| Miss Vickery, P.F. ... | 1 | 0 |
| E. C. Parker, P.F. ... | 1 | 0 |
| Isa Gilson, P.F. ... | 1 | 0 |
| M. A. Morgan, P.F. ... | 1 | 0 |
| S. M. Davis, P.F. ... | 1 | 0 |
| Mary Binkworth, P.F. | 1 | 0 |
| Nurse Pollock, P.F. ... | 1 | 0 |
| Sarah Newcomber, P.F. | 1 | 0 |
| Jane Burton, P.F. ... | 1 | 0 |
| E. Waller, P.F. ... | 1 | 0 |
| Policy 5,783, P.F. ... | 1 | 0 |
| S. A. Baxter, P.F. ... | 5 | 0 |
| Emma C. Briggs, P.F. | 5 | 0 |
| Nurse Parry ... ... | 5 | 0 |
| Nurse Barker ... ... | 5 | 0 |
| Nurse Dark, P.F. ... | 5 | 0 |
| Letitia W. Black, P.F. | 5 | 0 |
| Adeline E. C., P.F. ... | 5 | 0 |
| A. Whittome, P.F. ... | 5 | 0 |
| Mary Goodin, P.F., and K. F. A. Goodin, P.F. | 5 | 0 |
| Florence Still, P.F. ... | 5 | 0 |
| Policy 1,202, P.F. ... | 5 | 0 |
| S. Heathorn, P.F. ... | 5 | 0 |
| Ida J. Chaffe, P.F. ... | 5 | 0 |
| N. S. M. Allen, P.F.... | 5 | 0 |
| A. J. Gray ... | 5 | 0 |
| Mary Hicks, P.F. ... | 5 | 0 |
| E. Dynell Mayow, P.F. | 5 | 0 |
| Alice N. M. Newson, P.F. ... ... ... | 5 | 0 |
| Sarah Lane, P.F. ... | 5 | 0 |
| M. A. Winton, P.F. ... | 5 | 0 |
| Alice M. Priter, P.F... | 5 | 0 |
| Adelaide Bourne, P.F. | 5 | 0 |
| M. J. Dibble, P.F. ... | 5 | 0 |
| Nurse Cheek, P.F. ... | 5 | 0 |
| Nurse Briant, P.F. ... | 5 | 0 |
| M. M. Hunt, P.F. ... | 5 | 0 |
| F. C. Whitfield, P.F. | 5 | 0 |
| Nurse Cunningham, P.F. ... ... ... | 5 | 0 |
| M. J. Smith, P.F. ... | 5 | 0 |
| Nurse Oriel, P.F. ... | 5 | 0 |
| Miss Elizabeth Stanford, P.F. ... | 5 | 0 |
| M. A. Thornbers, P.F. | 5 | 0 |
| M. A. Jeffersons, P.F. | 5 | 0 |
| E. M. D. Walton, P.F. | 5 | 0 |
| Mary A. Barnes Groome, P.F. ... | 5 | 0 |
| Grace I. Walker, P.F. | 5 | 0 |
| Edith Ogden, P.F. ... | 5 | 0 |
| Nurse Hearne, P.F. ... | 5 | 0 |
| M. D. W. Ewing ... | 5 | 0 |
| F. Bickingham ... | 5 | 0 |
| Miss Hetherington, P.F.... ... | 5 | 0 |
| Policy 2,119 ... ... | 5 | 0 |
| J. Nabenn (?), P.F. ... | 5 | 0 |
| Nurse Brickmore, P.F. | 5 | 0 |
| E. E. Abram, P.F. ... | 5 | 0 |
| Nurse Weeberg ... | 5 | 0 |

| | s. | d. |
|---|---|---|
| Sister Marie Rees ... | 5 | 0 |
| M. A. Perman, P.F. ... | 5 | 0 |
| Nurse Platt, P.F. ... | 5 | 0 |
| Nurse Larkum, P.F. ... | 5 | 0 |
| Nurse Seaman Brown, P.F. ... ... ... | 4 | 0 |
| F. M. Hobbs, P.F. ... | 4 | 0 |
| L. Tudor, P.F. ... | 4 | 0 |
| C. B. Fairley, P.F., and H. Dickie, P.F. ... | 4 | 0 |
| A. Fricker, P.F. ... | 3 | 6 |
| A. Common, P.F. ... | 3 | 0 |
| Grace Smith, P.F. ... | 3 | 0 |
| M. C. Tarrant, P.F. ... | 3 | 0 |
| Margaret Dodd, P.F. | 3 | 0 |
| A Policy Holder, P.F. | 3 | 0 |
| Nurse Hamner, P.F.... | 3 | 0 |
| F. Hoare, P.F. ... | 3 | 0 |
| Miss Lambert, P.F. ... | 3 | 0 |
| Helen Payne, P.F. ... | 3 | 0 |
| Florence C. Gibbs, P.F. | 3 | 0 |
| Mary Pawyer, P.F. ... | 3 | 0 |
| A. McMillan, P.F. ... | 3 | 0 |
| Ellen Todd, P.F. ... | 3 | 0 |
| Georgina White, P.F. | 3 | 0 |
| E. Henry, P.F. ... | 3 | 0 |
| Louisa J. Evans, P.F. | 3 | 0 |
| Nurse Hanson... ... | 3 | 0 |
| E. Steward, P.F. ... | 2 | 6 |
| E. Bartholomew, P.F. | 2 | 6 |
| R. E. Curtis, P.F. ... | 2 | 6 |
| S. Buckley, P.F. ... | 2 | 6 |
| M. Johnson, P.F. ... | 2 | 6 |
| C. Connon, P.F. ... | 2 | 6 |
| Mary Barker, P.F. ... | 2 | 6 |
| H. L. Joad, P.F. ... | 2 | 6 |
| Mary M. Ogden, P.F. | 2 | 6 |
| L. A. Swift, P.F. ... | 2 | 6 |
| Eliza Babcock, P.F. ... | 2 | 6 |
| E. M. Manning, P.F... | 2 | 6 |
| Ada Holmes, P.F. ... | 2 | 6 |
| Amy L Pratt, P.F. ... | 2 | 6 |
| Lilian Cox, P.F. ... | 2 | 6 |
| Agnes Leah, P.F. ... | 2 | 6 |
| Kate Locker, P.F. ... | 2 | 6 |
| Emilie Nicholls, P.F... | 2 | 6 |
| S. E. Winter ... ... | 2 | 6 |
| Emma Lewis, P.F. ... | 2 | 6 |
| Sarah Wheatley, P.F. | 2 | 6 |
| Nurse Warren, P.F. ... | 2 | 6 |
| Nurse Nelson, P.F. ... | 2 | 6 |
| M. J. Ewart, P.F. ... | 2 | 6 |
| S. A. Sheldon, P.F. ... | 2 | 6 |
| Madame E. J. Laczkovie, P.F. ... | 2 | 6 |
| Alicia Trumble, P.F.... | 2 | 6 |
| Hester G. A. Harris, P.F. ... ... ... | 2 | 6 |
| Miss A. Collins ... | 2 | 6 |
| E. A. Edwards, P.F.... | 2 | 6 |
| Ida Ingram, P.F. ... | 2 | 6 |
| Margaret Smith, P.F. | 2 | 6 |
| Mrs. J. Chapman, P.F. | 2 | 6 |
| Louisa Pirouet, P.F. ... | 2 | 6 |
| Emma Hill, P.F. ... | 2 | 6 |
| Rachael Parsons, P.F... | 2 | 6 |
| Theodora Nicholson ... | 2 | 6 |
| Isobel C. Sherlock, P.F. | 2 | 6 |
| Nurse Simpson, P.F.... | 2 | 6 |
| R. Maunder, P.F. ... | 2 | 6 |
| S. Bardwell, P.F. ... | 2 | 6 |
| Mary Musgrave, P.F. | 2 | 6 |
| M. Mageem, P.F. ... | 2 | 6 |
| M. Martindale, P.F.... | 2 | 6 |
| E. A. Sheldon, P.F.... | 2 | 6 |
| A. C. Burnell, P.F. ... | 2 | 6 |
| E. Richmond, P.F. ... | 2 | 0 |
| Annie Ellis, P.F. ... | 2 | 0 |
| Nurse Routledge, P.F. | 2 | 0 |
| Nurse Bidwell, P.F. ... | 2 | 0 |
| M. Scott, P.F. ... | 2 | 0 |
| Teresa Keogh, P.F. ... | 2 | 0 |

| | s. | d. |
|---|---|---|
| A. Brindley, P.F. ... | 2 | 0 |
| Emily Scantlebury, P.F. | 2 | 0 |
| J. B. Marsh, P.F. ... | 2 | 0 |
| Louisa Hanch, P.F. ... | 2 | 0 |
| M. Pownall, P.F. ... | 2 | 0 |
| C. H. Grieg, P.F. ... | 2 | 0 |
| Melinda Smith, P.F.... | 2 | 0 |
| E. A. Vickers, P.F. ... | 2 | 0 |
| M. Bellamy, P.F. ... | 2 | 0 |
| M. J. Bailey, P.F. ... | 2 | 0 |
| Policy 533, P.F. ... | 2 | 0 |
| A. M. O Connor, P.F. | 2 | 0 |
| C. Marshall, P.F. ... | 2 | 0 |
| Nurse Ratcliffe, P.F.... | 2 | 0 |
| E. Marchant, P.F. ... | 2 | 0 |
| M. Maddison, P.F. ... | 2 | 0 |
| A. M. Pexton, P.F. ... | 2 | 0 |
| J. B. Baxter, P.F. ... | 2 | 0 |
| F. M. C. Lindsay, P.F. | 2 | 0 |
| J. Copley, P.F. ... | 2 | 0 |
| Theresa Brookes, P.F. | 2 | 0 |
| E. M. S. H. Clowes ... | 2 | 0 |
| A. E. Usher, P.F. ... | 2 | 0 |
| Nurse Frith, P.F. ... | 2 | 0 |
| Ruth E. Goodin, P.F. | 2 | 0 |
| J. Campbell, P.F. ... | 2 | 0 |
| Policy 547, P.F. ... | 2 | 0 |
| Policy 277, P.F. ... | 2 | 0 |
| E. Evans, P.F. ... | 2 | 0 |
| J. Rochester, P.F. ... | 2 | 0 |
| Nurse Coy, P.F. ... | 2 | 0 |
| Emily Graham ... | 2 | 0 |
| Lavinia Frankes, P.F. | 2 | 0 |
| A. Cosgrave, P.F. ... | 2 | 0 |
| C. Ann Davidson, P.F. | 2 | 0 |
| E. A. Taylor, P.F. ... | 2 | 0 |
| M. A. Upton, P.F. ... | 2 | 0 |
| Nurse Watson, P.F. ... | 2 | 0 |
| Nurse Bray, P.F. ... | 2 | 0 |
| R. McKenna, P.F. ... | 2 | 0 |
| Gertrude Sothern (?), P.F. ... ... ... | 2 | 0 |
| E. Cooling, P.F. ... | 1 | 6 |
| S. E. Love, P.F. ... | 1 | 6 |
| C. M. Symonds, P.F. | 1 | 6 |
| H. Haynes, P.F. ... | 1 | 6 |
| B. Bloomer, P.F. ... | 1 | 6 |
| E. A. Hudson Cox, P.F. | 1 | 0 |
| C. C. Porter, P.F. ... | 1 | 0 |
| Annie Bryan, P.F. ... | 1 | 0 |
| C. E. Baker, P.F. ... | 1 | 0 |
| L. H. Wilson, P.F. ... | 1 | 0 |
| C. Wintertone, P.F. ... | 1 | 0 |
| S. F. Dudley, P.F. ... | 1 | 0 |
| M. S. Rees ... ... | 1 | 0 |
| P. A. Milne, P.F. ... | 1 | 0 |
| M. Turner, P.F. ... | 1 | 0 |
| Rosa E. Bennion, P.F. | 1 | 0 |
| Edith M. Plomley, P.F. | 1 | 0 |
| M. Haughton, P.F. ... | 1 | 0 |
| S. A. Wills, P.F. ... | 1 | 0 |
| C. E. Spauholtz, P.F. | 1 | 0 |
| C. Tomlinson, P.F. ... | 1 | 0 |
| Nurse Sharpe, P.F. ... | 1 | 0 |
| Mary Baker, P.F. ... | 1 | 0 |
| Maria Church, P.F. ... | 1 | 0 |
| L. Carter, P.F. ... | 1 | 0 |
| Emily G. Loocker, P.F. | 1 | 0 |
| E. E. Drake, P.F. ... | 1 | 0 |
| Edith B. Cornwall, P.F. | 1 | 0 |
| Nurse Pearce, P.F. ... | 1 | 0 |
| Nurse Wilson, P.F. ... | 1 | 0 |
| M. Tozer ... ... | 1 | 0 |
| Nurse Bartlett, P.F. ... | 1 | 0 |
| Nurse England, P.F.... | 1 | 0 |
| Miss J. Langley, P.F. | 1 | 0 |
| M. S. Pratt, P.F. ... | 1 | 0 |
| E. M. Wilson, P.F. ... | 1 | 0 |
| Francis E. Driver, P.F. | 1 | 0 |
| S. Helen Flude, P.F.... | 1 | 0 |
| E. Gillingham, P.F. ... | 1 | 0 |
| Policy 5,154, P.F. ... | 1 | 0 |

| | s. | d. |
|---|---|---|
| Jane Lawrence, P.F.... | 1 | 0 |
| E. C. Stevens, P.F ... | 1 | 0 |
| Alice Greening, P.F.... | 1 | 0 |
| Annie Machie, P.F. ... | 1 | 0 |
| Agnes Ellen Fairman, P.F. ... ... ... | 1 | 0 |
| M. A. Mackie, P.F. ... | 1 | 0 |
| E. M. Tubbs, P.F. ... | 1 | 0 |
| M. A. Leppey, P.F. ... | 1 | 0 |
| Nurse Cecil ... ... | 1 | 0 |
| Annie Clay, P.F. ... | 1 | 0 |
| R. G. S. Campbell, P.F. | 1 | 0 |
| Isabel Biggins, P.F. ... | 1 | 0 |
| S. J. Butcher, P.F. ... | 1 | 0 |
| Mrs. E. M. Thornton, P.F. ... ... ... | 1 | 0 |
| Esther Edgem, P.F. ... | 5 | 0 |
| A Nurse at Hyeres ... | 5 | 0 |
| E. M. Cunningham, P.F. ... ... ... | 5 | 0 |
| Alice Radford... ... | 5 | 0 |
| Mary E. Vasey, P.F... | 5 | 0 |
| J. Riddock, P.F. ... | 5 | 0 |
| E. Winton, P.F. ... | 5 | 0 |
| M. J. Butcher, P.F. ... | 5 | 0 |
| F. M. Hadley... ... | 5 | 0 |
| B. L. Colborne, P.F.... | 5 | 0 |
| Miss E. A. Wildman, P.F. ... ... ... | 5 | 0 |
| Miss Shute, P.F. ... | 5 | 0 |
| Jessie Brigham, P.F... | 5 | 0 |
| F. Lewis, P.F... ... | 5 | 0 |
| E. J. Allard, P.F. ... | 5 | 0 |
| Miss Kent, P.F. ... | 5 | 0 |
| J. Bowman, P.F. ... | 5 | 0 |
| S. S. S. Lodget, P.F ... | 5 | 0 |
| Policy 4,752 (E. G. Benwell), P.F. ... | 5 | 0 |
| M. E. Cole, P.F. ... | 5 | 0 |
| M. S. Barwell... ... | 5 | 0 |
| S. A. Elmer, P.F. ... | 5 | 0 |
| Florence J. A. Coates, P.F. ... ... ... | 5 | 0 |
| E. Payn, P.F.... ... | 5 | 0 |
| E. Halliday, P.F. ... | 5 | 0 |
| Nurse Swift, P.F. ... | 5 | 0 |
| C. Harcourt, P.F. ... | 5 | 0 |
| Mary Dimsdale, P.F.... | 5 | 0 |
| Adelaide Spurling, P.F. | 5 | 0 |
| C. Iliffe Harrison, P.F. | 5 | 0 |
| M. Hart, P.F.... ... | 5 | 0 |
| A. E. Briggs, P.F. ... | 5 | 0 |
| E. Jones, P.F. ... | 5 | 0 |
| C. Newman, P.F. ... | 5 | 0 |
| H. Gray, P.F.... ... | 4 | 0 |
| A. Young, P.F., and E. E. Young, P.F.... | 4 | 0 |
| C. E. Jenks, P.F. ... | 4 | 0 |
| W. A. Titchetts, P.F. | 4 | 0 |
| M. A. Parkett, P.F. ... | 3 | 6 |
| Nurse Lovell, P.F. ... | 3 | 0 |
| Anne Jordan, P.F. ... | 3 | 0 |
| Marie Stefansen ... | 3 | 0 |
| Ada Scott, P.F. ... | 3 | 0 |
| Nurse Bates, P.F. ... | 3 | 0 |
| Mary J. Carter, P.F... | 3 | 0 |
| K. E. Stannett, P.F... | 3 | 0 |
| Margaret M. E. Hay, P.F. ... ... ... | 3 | 0 |
| Nurse Leedham, P.F... | 3 | 0 |
| Nurse Jacques, P.F. ... | 2 | 6 |
| Nurse March, P.F. ... | 2 | 6 |
| Amy Brown, P.F. ... | 2 | 6 |
| B. C. Drake, P.F. ... | 2 | 6 |
| Mrs. Lee, P.F. ... | 2 | 6 |
| L. L. Ward, P F. ... | 2 | 6 |
| Alice Herlst, P.F. ... | 2 | 6 |
| Margaret Graham, P.F. | 2 | 6 |
| Miss Lindsay, P.F. ... | 2 | 6 |
| Helen M. Coleman, P.F. | 2 | 6 |
| Mary O'Brien ... ... | 2 | 6 |
| Policy 4,378, P.F. ... | 2 | 6 |

| | s. | d. |
|---|---|---|
| Mary E. Jennings, P.F. | 2 | 6 |
| E. Craggs, P.F. ... | 2 | 6 |
| A. Ovard, P.F. ... | 2 | 6 |
| M. E. Spread, P.F. ... | 2 | 6 |
| M. Pratt, P.F. ... | 2 | 6 |
| S. Cudlipp, P.F. ... | 2 | 6 |
| M. Webb ... ... | 2 | 6 |
| M. Edwards, P.F. ... | 2 | 6 |
| S. A. Reynolds, P.F.... | 2 | 6 |
| M. G. A. Warner, P.F. | 2 | 6 |
| Miss Hooper, P.F. ... | 2 | 6 |
| C. S. Read, P.F. ... | 2 | 6 |
| E. Burton, P.F. ... | 2 | 6 |
| A. L. Pollard, P.F. ... | 2 | 6 |
| M. A. Edwards, P.F. | 2 | 6 |
| S. J. Smith, P.F. ... | 2 | 6 |
| Cth. E. Redding, P.F. | 2 | 6 |
| Nurse Shepherd, P.F. | 2 | 6 |
| E. A. Cozens, P.F. ... | 2 | 6 |
| Nurse Simmons, P.F. | 2 | 6 |
| A. F. Overton, P.F.... | 2 | 0 |
| Nurse Nottage, P.F.... | 2 | 0 |
| Emily A. Oliver, P.F. | 2 | 0 |
| Nurse Ruddefoot, P.F. | 2 | 0 |
| Margaret Lindsay, P.F. | 2 | 0 |
| M. Berwick, P.F. ... | 2 | 0 |
| Alice L. Von Dix, P.F. | 2 | 0 |
| E. Thorn, P.F. ... | 2 | 0 |
| Mrs. Nicholson, P.F... | 2 | 0 |
| T. R. Walker, P.F. ... | 2 | 0 |
| E. Moore, P.F. ... | 2 | 0 |
| Emma Bayliss ... | 2 | 0 |
| Alice Jones, P.F. ... | 2 | 0 |
| C. E. Walker, P.F. ... | 2 | 0 |
| H. A. Chaplin, P.F. ... | 2 | 0 |
| Emma Welch, P.F. ... | 2 | 0 |
| M. Shefford, P.F. ... | 2 | 0 |
| E. Katie Sharp, P.F... | 2 | 0 |
| Florence Ratcliffe, P.F., and Jessie Ratliff, P.F. ... ... ... | 2 | 0 |
| Emma R. Turner, P.F. | 2 | 0 |
| Nurse Friend, P.F. ... | 2 | 0 |
| Mary A. Stroud, P.F. | 2 | 0 |
| E. Sherburn, P.F. ... | 2 | 0 |
| C. A. Cooling, P.F. ... | 2 | 0 |
| E. Porter, P.F. ... | 2 | 0 |
| E. Johnston, P.F. ... | 2 | 0 |
| J. Turner, P.F. ... | 2 | 0 |
| Sarah Hodgson, P.F... | 2 | 0 |
| J. Stevens ... ... | 2 | 0 |
| Mary A. Rook, P.F.... | 2 | 0 |
| Emily Lynes, P.F. ... | 2 | 0 |
| Alice M. Olivery, P.F. | 2 | 0 |
| Gertrude Pike, P.F.... | 1 | 6 |
| Nurse Hodge, P.F. ... | 1 | 6 |
| Policy Holder, P.F. ... | 1 | 6 |
| Nurse Steele, P.F. ... | 1 | 3 |
| K. Rogers, P.F. ... | 1 | 0 |
| Nurse Brown, P.F. ... | 1 | 0 |
| M. Williams, P.F. ... | 1 | 0 |
| M. Langley, P.F. ... | 1 | 0 |
| Elizabeth Bishop, P.F. | 1 | 0 |
| Nellie P. Simpleton, P.F. | 1 | 0 |
| E. B. Boddington, P.F. | 1 | 0 |
| L. Badger, P.F. ... | 1 | 0 |
| Harriet Brown, P.F ... | 1 | 0 |
| A. Evelyn Smith, P.F. | 1 | 0 |
| E. A. Fenton, P.F. ... | 1 | 0 |
| M. A. Bryan, P.F. ... | 1 | 0 |
| E. Hackett, P.F. ... | 1 | 0 |
| Nurse Carmen, P F. ... | 1 | 0 |
| A. C. Boofish, P.F. ... | 1 | 0 |
| E. Keen, P F.... ... | 1 | 0 |
| C.L E. Orgelmann, P.F. | 1 | 0 |
| Fanny Feddarb, P F... | 1 | 0 |
| Nurse Kynaston, P.P. | 1 | 0 |
| Nurse Nicklin, P.F. ... | 1 | 0 |
| Policy 4,148 ... ... | 1 | 0 |
| E. Christy, P.F. ... | 1 | 0 |
| Nurse Fricker, P.F. ... | 1 | [illegible] |
| S. Jane Holden, P.F.... | 1 | [illegible] |

| | s. | d. |
|---|---|---|
| Emily F. Groome, P.F. | 1 | 0 |
| Mary E. Waltham, P.F. | 1 | 0 |
| W. Upham, P.F. ... | 1 | 0 |
| C. S. Phillips, P.F. ... | 1 | 0 |
| H. Tykes, P.F. ... | 1 | 0 |
| Kate Creeds, P.F. ... | 1 | 0 |
| Miss E. Roseblede, P.F. | 1 | 0 |
| Janet Hare, P.F. ... | 1 | 0 |
| Jane Edwards, P.F. ... | 1 | 0 |
| Margaret Hewat ... | 1 | 0 |
| M. J. Kyte, P.F. ... | 1 | 0 |
| Mrs. Amy Potts, P.F. | 1 | 0 |
| Nurse Pierce, P.F. ... | 1 | 0 |
| H. Elsley, P.F. ... | 1 | 0 |
| Jessie E. Solly, P.F. ... | 1 | 0 |
| M. S. Hodgson, P.F.... | 1 | 0 |
| Maria Burford, P.F. ... | 1 | 0 |
| S. Castlecote, P.F. ... | 5 | 0 |
| Catherine Tunaly, P.F. | 5 | 0 |
| E. A. Phillips, P.F. ... | 5 | 0 |
| Francis Laing, P.F. ... | 5 | 6 |
| S. A. Baker, P.F. ... | 5 | 0 |
| Elsie Whitaker, P.F.... | 5 | 0 |
| Annie Lawson, P.F. ... | 5 | 0 |
| Nurse Cunningham ... | 5 | 0 |
| Sister Julia, P.F. ... | 5 | 0 |
| Jane Sinclair, P.F. ... | 5 | 0 |
| E. M. C. Gray, P.F.... | 5 | 0 |
| Edith L. S. Sago, P.F. | 5 | 0 |
| Lucy Gunn, P.F. ... | 5 | 0 |
| S. E. Lovatt, P.F. ... | 5 | 0 |
| S. E. Harrison, P.F.... | 5 | 0 |
| Nurse Parker, P.F. ... | 5 | 0 |
| Miss Chapman, P.F. ... | 5 | 0 |
| M. Lohman, P.F. ... | 5 | 0 |
| S. Pitchgood, P.F. ... | 5 | 0 |
| Eleanor Clarke, P.F.... | 5 | 0 |
| J. E. Edwards, P.F. ... | 4 | 6 |
| C. M. Ballard, P.F. ... | 3 | 6 |
| J. Nash, P.F.... ... | 3 | 0 |
| Nurse Element, P.F.... | 3 | 0 |
| M. J. Seaward, P.F.... | 3 | 0 |
| A. Cannon, P.F. ... | 3 | 0 |
| Florence Holmes, F.F. | 3 | 0 |
| Policy 6,956, P.F. ... | 2 | 6 |
| Policy 3,070, P.F. ... | 2 | 6 |
| M. L. Smith, P.F. ... | 2 | 6 |
| S. D. Rolls, P.F. ... | 2 | 6 |
| Nurse Rogerson ... | 2 | 6 |
| Nurse Bloomfield, P.F. | 2 | 6 |
| Elizabeth Willott, P.F. | 2 | 6 |
| Helen Pugh, P.F. ... | 2 | 6 |
| Emma Brockway, P.F. | 2 | 6 |
| C. Newsome, P.F. ... | 2 | 6 |
| Mary E. Taylor, P.F. | 2 | 6 |
| M. T. Walmsley, P.F. | 2 | 6 |
| Nurse Clay, P.F. ... | 2 | 6 |
| A. Maxfield, P.F. ... | 2 | 6 |
| M.E. MacDo[illegible], P.F. | 2 | 6 |
| Nurse Chippendale, P.F. | 2 | 6 |
| A. Bats, P.F. ... ... | 2 | 6 |
| Mary A. Adkins, P.F. | 2 | 6 |
| Miss Prentice, P.F. ... | 2 | 6 |
| Jane Spragge, P.F. ... | 2 | 6 |
| Eliza Wycherley, P.F. | 2 | 6 |
| Helen M. Patterson ... | 2 | 6 |
| J. Gregorson, P.F. ... | 2 | 6 |
| Nurse Wright, P.F. ... | 2 | 6 |
| Mary Parratt, P.F. ... | 2 | 6 |
| A. Beer, P.F.... ... | 2 | 6 |
| M. Lawrence, P.F. ... | 2 | 6 |
| E. Pettifer ... ... | 2 | 6 |
| A. Pettifer ... ... | 2 | 6 |
| Nurse Logie ... ... | 2 | 6 |
| Nurse Williamson ... | 2 | 6 |
| L. Wills ... ... | 2 | 0 |
| Nurse Kerr ... ... | 2 | 0 |
| Nurse S. Walker, P.F. | 2 | 0 |
| A. A. Nicholas, P.F.... | 2 | 0 |
| Beatrice Churchill, P F. | 2 | 0 |
| Elizabeth Hitchings, P.F. ... ... ... | 2 | 0 |

| | s. | d. |
|---|---|---|
| Nurse Coleway, P.F... | 2 | 0 |
| E. B. Fredericks, P.F. | 2 | 0 |
| N. Nacer, P.F. ... | 2 | 0 |
| Gertrude Dunn, P.F.... | 2 | 0 |
| Gertrude, P.F. ... | 2 | 0 |
| Alice Blomfield, P.F.... | 2 | 0 |
| Jane Galwan, P.F. ... | 2 | 0 |
| A. Chaloner, P.F. ... | 2 | 0 |
| Jane Barlow, P.F. ... | 2 | 0 |
| Nurse Borgin, P.F. ... | 2 | 0 |
| E. Williamson, P.F. ... | 2 | 0 |
| Nurse Cherry and Nurse Yeoman ... | 2 | 0 |
| Nellie Lee ... ... | 2 | 0 |
| A District Nurse ... | 2 | 0 |
| Nurse Margetts, P.F... | 2 | 0 |
| C. J. Peacock, P.F. ... | 2 | 0 |
| Nurse Oliver, P.F. ... | 2 | 0 |
| Maria Jones, P.F. ... | 2 | 0 |
| Florence Puddicombe, P.F. ... ... ... | 2 | 0 |
| Nurse Marsh, P.F. ... | 2 | 0 |
| Nurse Thaxter, P.F. ... | 2 | 0 |
| Nurse ... ... ... | 2 | 0 |
| Nurse Thompson, P.F. | 2 | 0 |
| K. S. Bulteel, P.F. ... | 2 | 6 |
| Nurse Sylvia, P.F. ... | 1 | 6 |
| Nurse Weymouth, P.F. | 1 | 6 |
| L. Banbrook, P.F ... | 1 | 6 |
| Nurse Dow ... ... | 1 | 0 |
| M. Beavan, P.F. ... | 1 | 0 |
| M. Sanders, P.F. ... | 1 | 0 |
| E. Ward, P.F. ... | 1 | 0 |
| E. J. Beswick, P.F. ... | 1 | 0 |
| P. Mills, P.F. ... ... | 1 | 0 |
| E. Jeffrey, P.F. ... | 1 | 0 |
| E. S. Whitchurch, P.F. | 1 | 0 |
| L. Barnwell, P.F. ... | 1 | 0 |
| Dorothy E. Hawkins, P.F. ... ... ... | 1 | 0 |
| Louisa Nelhams, P.F. | 1 | 0 |
| E. S. Clarke, P.F. ... | 1 | 0 |
| Nurse Theobald, P.F. | 1 | 0 |
| Lucy A. Bacon, P.F. | 1 | 0 |
| M. A. Cassady, P.F. | 1 | 0 |
| Miss A. Huntley, P.F. | 1 | 0 |
| Miranda Thomas, P.F. | 1 | 0 |
| A. Hird, P.F. ... ... | 1 | 0 |
| A. C. Way, P.F. ... | 1 | 0 |
| Miss Saun[illegible]er, P.F. ... | 1 | 0 |
| C. Wood, P.F... ... | 1 | 0 |
| Miss Carruthers, P.F... | 1 | 0 |
| Nurse Ellen, P.F. ... | 1 | 0 |
| J. Parker, P.F... ... | 1 | 0 |
| Policy 5,857, P.F. ... | 1 | 0 |
| Mary Smith, H.F. ... | 1 | 0 |
| M. E. Walker... ... | 1 | 0 |
| M. M. Buxton, P.F.... | 1 | 0 |
| J. Pugh, P.F.... ... | 5 | 0 |
| R. Florence, P.F. ... | 5 | 0 |
| Mary E. Rudd, P.F.... | 5 | 0 |
| Margaret Coffey, P.F. | 5 | 0 |
| M. Lobb, P.F... ... | 5 | 0 |
| E. M. A. Head, P.F... | 5 | 0 |
| Emily Lucas, P.F. ... | 5 | 0 |
| Mary Shepherd ... | 5 | 0 |
| Cecelia Fallon, P.F. ... | 5 | 0 |
| Margaret Bowles, P.F. | 5 | 0 |
| Anna Hook, P.F. ... | 5 | 0 |
| Annie M. Breaker, P.F. | 5 | 0 |
| E. B. Jobson, P.F. ... | 5 | 0 |
| Miss Peter, P.F. ... | 5 | 0 |
| Margaret J. Brewster, P.F. ... ... ... | 5 | 0 |
| Nurse Barker, P.F. ... | 5 | 0 |
| Anonymous ... ... | 5 | 0 |
| Miss Corneille, P.F. ... | 5 | 0 |
| Eliza Rochester, P.F... | 5 | 0 |
| Rosa Annie Trow, P.F. | 5 | 0 |
| F. E. L. Peirce ... | 5 | 0 |
| Whinbush ... ... | 5 | 0 |
| F. A. Williams, P.F... | 5 | [illegible] |

### Everybody's Opinion.

[Correspondence on all subjects is invited, but we cannot in any way be responsible for the opinions expressed by our correspondents. No communication can be entertained if the name and address of the correspondent is not given, as a guarantee of good faith but not necessarily for publication, or unless one side of the paper only is written on.]

### OUR CLOTHING DISTRIBUTION.

The Matron of the Royal Hospital for Children and Women, Waterloo Bridge Road, writes: I thank you for so kindly sending clothing for the patients here; they will be most useful.

The Secretary, Charing Cross Hospital, writes: I am directed by the board of governors to convey to you their sincere thanks for your kind present of clothing for the patients.

The Lady Superintendent of the West Ham Hospital, E., writes: I thank you very much for the parcel you have sent us from your Hospital Clothing Fund. The garments are very welcome in this hospital, as all our patients are poor and have need of them.

The Matron of the West London Hospital, Hammersmith Road, writes: I beg to return you many thanks for your kind present of gifts for the patients in this hospital. They will be much appreciated.

The Matron of King's College Hospital, W.C., writes: Will you accept our most grateful thanks for the kind gift of clothes from patients this Christmastide, a very welcome gift, and one much valued by the sick and ourselves.

The Matron of St. Marylebone Infirmary, North Kensington, W., writes: I am much obliged for the parcel of warm clothing sent for the patients, which will be much appreciated by them.

### UNTRAINED NURSES.

"Nurse Katharine" writes: Though "C. R." has much to be annoyed at, I sincerely hope she will not put all maternity nurses in the same category with the two unscrupulous ones she mentions. I am a maternity nurse myself, having been trained in a good school and taken my L.O.S. certificate, but, though I have often been asked to do so, I should not dream of taking medical and surgical cases, nor do I think would any conscientious maternity nurse unless in an emergency. To begin with, I have as much of my own work as I can manage, and if I had not, setting conscientious scruples aside, I should never, for my own credit's sake, undertake any case I did not feel thoroughly competent to nurse. The matter of temperatures can surely not refer to us? For anyone who knows what maternity nursing is must realise the great importance of accurate pulse and temperature taking. As for the doctor referred to, I can only imagine that his knowledge of trained nurses was very slight, or he could never have so unblushingly declared that "few know a clinical thermometer!" I sincerely pity the poor man, and hope that in future he will get his nurses from some good institution or hospital, or else learn to recognise the difference between an "impostor" and a "trained" nurse.

### DISTRICT NURSING.

"A District Nurse of Four-and-a-Half Years Standing" writes: I would advise the nurse anxious to have her own cottage not to undertake so much unnecessary work. The result would be a breakdown sooner or later. The strain would be too great, unless a friend could bear the brunt of cooking and housework. How can justice be done to the patients if the nurse has all to do for herself in her free time? She cannot rest, as there is always, even with only two rooms, some dusting, cleaning or dish-washing, to do in the time she should be resting or taking recreation. A woman once a week for a general turn-out cannot keep a place decent for the remaining six days. Funds will not permit of daily help. Imagine the misery on a cold winter morning of having to prepare your own breakfast before a good foundation can be laid for a day in the district. The dinner, too, cannot always be a cold one, and must be prepared before commencing duty. Gas or oil, of course, may be used; but how much more satisfied with self and the world in general will the nurse be who has had a good warm before turning out? A nurse may be as strong as a horse, but either her health or her temper—possibly and very probably both—will suffer if she attempt the charge of a district and her own household cares at the same time. I have heard of a district nurse whose kind landlady would give no fire for early morning; of another who could get neither puddings nor hot water without much grumbling. Still, as a rule, much may be done with a little tact. Some grievance, as a rule, has to be borne in all work. Surely it is best not to sacrifice health.

### A FEW HINTS FOR THE PRIVATE NURSING OF TYPHOID.

Sister Neale writes: I feel it is my duty to write and contradict the letter that appeared in last week's Hospital; there were so many things in it that were not correct, *i.e.*: The poison which conveys the disease is abundantly contained in the motion. It is absolutely necessary to disinfect these. After removing bed-pan from the patient, with 1 in 20 carbolic solution, it is wiser to use old linen instead of paper and then burn immediately, disinfect drain after emptying the motion with 1 in 20 carbolic. The stool should be emptied immediately, providing the doctor has already ascertained its character, not keep it for two or three hours, as the nurse tells us; it is after the motion has been standing long that there is danger to the nurse. I cannot help thinking that the nurse knows very little about the nursing of typhoid. I will give a few hints from my experience. Always boil the milk yourself and strain it, give barley water in preference to soda water, if there is vomiting mix lime water with the milk, and feed the patient at stated intervals; keep written report of everything; take the temperature pulse and respiration every four hours. Guard against inhaling the patient's breath, and never swallow saliva after emptying the bed-pan; always wash your hands and disinfect them before leaving the room. If these few hints were carried out strictly there would be fewer youthful nurses attacked with the disease.

*** We print the above somewhat curious letter on the general principle of letting everyone have her say. At the same time we would suggest to all our correspondents—whose communications we are always glad to insert—that in criticising the opinions of others it is wise not to be too positive about one's own, and that above all things gentleness of language is a virtue. Why should Sister Neale gratuitously go out of her way to say that the nurse whose writings she criticises "knows very little about the nursing of typhoid," and why should she feel it her "duty to write and contradict the letter that appeared in last week's Hospital, there were so many things in it which were not correct," when, after all, the only thing which she points out in the whole letter in regard to which she differs is as to the treatment of the evacuations? Let us urge all our correspondents to be gentle with each other. As to the particular question at issue, we do not want to judge between "Sister" and "Nurse," but at least we may point out that, in urging that the motions should be disinfected with 1 in 20 carbolic solution, Sister Neale is but repeating the advice of "A Private Nurse," who had already said "pour a strong solution of carbolic over it, cover with a cloth wrung out of carbolic lotion, and keep for the doctor's inspection, or for two or three hours before emptying down the drain." Between "strong solution" and "1 in 20 solution" there is not much to choose. The great point of difference between these two writers, the difference on the strength of which Sister Neale is drawn to say that she "cannot help thinking that the nurse knows very little about the nursing of typhoid," is in regard to the question whether to pour the evacuations down the drain "immediately," or to keep them in carbolic for a couple of hours first. Sister Neale seems entirely to have missed the point that disinfection takes time. To say that "it is absolutely necessary to disinfect" the stool, and then to say that "the stool should be emptied immediately" is a contradiction in terms. Some authorities advise that the evacuation should be left in the disinfectant for twenty minutes, and some for an hour; "A Private Nurse" says two hours, and many who know most about the matter are inclined very much to doubt whether under ordinary circumstances household disinfection by mixture with chemical solutions is ever really effective. But no one, we fancy, believes that it is possible to disinfect immediately, any more than that it is possible to "disinfect the drain, after emptying the motion, with 1 in 20 carbolic." Our own opinion is in favour of the proceeding recommended by Sister Neale, namely that when there is a good drain one should get the stuff down it as quickly as possible; but we base that opinion largely upon our scepticism as to the practical possibility of obtaining a really efficient disinfection of the stools by any such method as is suggested. There can be no doubt that if disinfection is what is aimed at, which is Sister Neale's point, this is much more likely to be attained by the method advocated by "A Private Nurse," than by the immediate emptying advocated by Sister Neale.—ED. *T. H.*

### For Reading to the Sick.

### THE NEW YEAR.

"CHOOSE you this day whom ye will serve."—*Josh.* xxiv. 15.

"Forgetting those things that are behind and reaching forth unto those things that are before, I press toward the mark for the prize of the high calling."—*Phil.* iii. 14.

The year departs! a blessing on its head!
We mourn not for it, for it is not dead:
Dead? What is that? A word to joy unknown,
Which love abhors, and faith will never own.
The passing breezes gone as soon as felt,
The flakes of snow that in the soft air melt,
The smile that sinks into a maiden's eye,
They come, they go, they change, they do not die.
So the Old Year—that fond and formal name—
Is with us yet—another and the same.
And are the thoughts that evermore are fleeing,
The moments that make up our being's being,
The silent workings of unconscious love
Or the dull hate which clings and will not move,
Are these less vital than the wave or wind
Or snow that melts and leaves no trace behind?
—*H. Coleridge.*

You need the lower life to stand upon
In order to reach up unto that higher;
And none can stand a tip-toe in the place
He cannot stand in with two stable feet.
—*E. B. Browning.*

**Reading.**

Experience itself will show you, if you will make trial of it, that the path of charity and love towards God and our neighbour is the most clear and plain road which leads to eternal life.

Let everything be a means of leading you to God, and let nothing detain you on the way. Let all your labours be directed to your Lord; love Him, and give to Him your whole heart without any fear; for He will find a good way to solve all your doubts, and will restore you when you fall. Lastly, in one word, if you will love Him, you shall possess all good. Offer yourself to God for a sacrifice, in peace and quietness of spirit. And the better to advance in this journey, and to bear yourself up without weariness and vexation, you should at every step dispose your soul by expanding your will to the extent of the Divine Will; and the more your will is thus expanded, the more you will receive. —*Senpoli.*

Let us never be afraid of innocent joy. God is good, and what He does is well done; resign yourself to everything, even to happiness; ask for the spirit of sacrifice, of detachment, of renunciation, and, above all, for the spirit of joy and gratitude—that genuine and religious optimism which sees in God a father, and asks no pardon for His benefits. We must dare to be happy, and dare to confess it, regarding ourselves always as the depositaries, not as the authors of our own joy.—*Amiel.*

It is one of the blessed uses of the life of an earthly home, under the power of the grace of God, to help the soul to conquer sin, to rise above the heartburnings and misconstructions, to watch against careless or culpable mistakes, to grow in strength and learn that strength is a duty, to shun temptation or to conquer it, and to spread the peace of God with its inward sweetness and outward cheerfulness over other lives.—*Knox-Little.*

Go where thou wilt, seek whatsoever thou wilt, thou shalt not find a higher way above, nor a safer way below, than the way of the Holy Cross.—*Thos. à Kempis.*

### Notes and Queries.

**The contents of the Editor's Letter-box have now reached such unwieldy proportions that it has become necessary to establish a hard and fast rule regarding Answers to Correspondents. In future, all questions requiring replies will continue to be answered in this column without any fee. If an answer is required by letter, a fee of half-a-crown must be enclosed with the note containing the enquiry. We are always pleased to help our numerous correspondents to the fullest extent, and we can trust them to sympathise in the overwhelming amount of writing which makes the new rules a necessity.**

**Every communication must be accompanied by the writer's name and address, otherwise it will receive no attention.**

*Tubercular.*

(134) Will you kindly tell me of a home or hospital in or near London where a boy of 14, who suffers from tubercular abscesses and is unable to walk, could be received for proper treatment? Some payment could be made.—*Verena.*

The case seems suitable for any general hospital.

*Training.*

(135) Would you kindly give me some information about a suitable hospital or nursing home in Liverpool or London where I could go for three or six months' training, and what terms (moderate)?—*M. Owen.*

See "The Nursing Profession: How and Where to Train," price 2s., from the Scientific Press.

*Mental Nursing.*

(136) I am a doctor's wife, and have had much experience in mental cases. Owing to my husband developing mental trouble, I have had to give up my house, but before that, and during the last twelve years, we had mental cases in our own house, both under commissioners and otherwise. I am very anxious to obtain employment in an asylum, and write to ask if you think I am suitable. I am 35 years of age, medium height, strong, and very active, well educated, and refined. I can give good references.—*R. R.*

You seem well adapted for work with the mentally afflicted. A short advertisement will probably bring you a good number of replies.

*Rheumatic Fever.*

(137) Would the Editor kindly inform "E. H." if Norwood would be a suitable place for a patient recently recovered from rheumatic fever to live in, or would the clay soil be a decided objection if the house was in a high position?

A clay soil is generally objectionable where there is a tendency to rheumatism; but much depends upon how far a district is covered with houses. Country clay is bad, but where clay soil is well drained and a large proportion is covered with roofs and weil-paved streets and yards, it is healthy enough.

*Massage Certificate.*

(138) Can you tell me which is supposed to be the best certificate for a masseuse to hold, and how it is to be obtained?—*S. M.*

That of the Society of Trained Masseuses, 12, Buckingham Street, Strand, W.C. Write to the secretary of the society for further information.

*L'Institut Longcott.*

(139) Will you kindly tell me whether L'Institut Longcott really exists, and whether instruments for deafness are given away gratis?—*E. R. W.*

Deafness is not a matter to be trifled with. Consult a medical man. Yes. Certain patent ear drums are sold by this institute. The advice is free by post, but the drums are expensive. May we point out that it is not often possible to diagnose the cause of deafness without an exceedingly careful personal examination, and that, too, by a specialist?

*Seborrhœa.*

(140) Will you or your numerous correspondents kindly tell me what I should use for the treatment of seborrhœa? It is a sequel to eczema on the head not properly attended to in childhood. The great inconvenience is the loss of hair and greasiness of the skin. I see the subject was only touched upon in THE HOSPITAL a short time back.—*Perplexed.*

Consult a specialist. There is an article in THE HOSPITAL for July 31st, 1897, which goe very fully into the treatment of this disease. It is entitled "Dandruff and Baldness."

*Christmas Day.*

(141) Can the Editor of THE HOSPITAL tell "L. W." in which year Christmas Day last fell on a Monday?

Six years ago; in 1893.

*Carbolic Acid.*

(142) Would you kindly tell me what would be a good and cheap carbolic to use for disinfecting linen? I have tried No. 5 Calvert's Carbolic. This mixes very well in a 1 in 80 solution (as directed on the bottles), but it will not properly mix in a 1 in 40 solution. I am using a good quantity, and the purer kind is so expensive.—*E. C. McGill.*

Calvert's is certainly the most convenient and the cheapest. Why not, if it be too expensive, use a cheaper disinfectant such as chloride of lime? If carefully and properly used the chloride of lime bleaches the linen and does not destroy it more than other chemicals; but boiling is the best mode of disinfecting linen.

*Uniform.*

(143) Will you kindly tell me whether a lady nurse (not trained) to young children ever wears uniform?—*E. M. B.*

The wearing of uniform is merely a matter of private arrangement. A child's nurse ought not to wear the dress of a sick nurse, as it is misleading; nevertheless many of them do so.

*Crutch and Kindness League.*

(144) The address of the Crutch and Kindness League asked for in "Notes and Queries" is Mr. John Kirk, Ragged School Union, 37, Norfolk Street, Strand, W.C.—*F. E. S.*

## Figures and Tables

**Figure f1:**
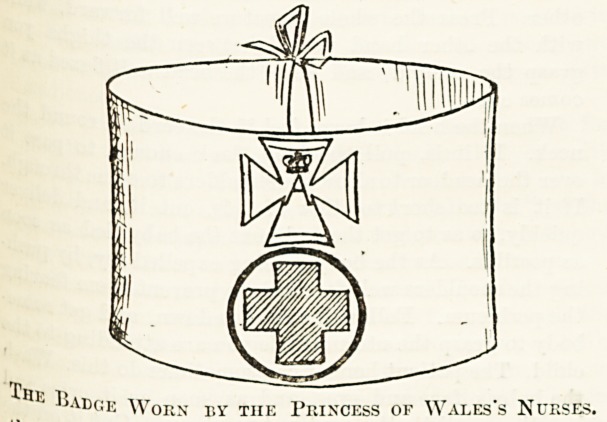


**Figure f2:**